# Materials A^II^LnInO_4_ with Ruddlesden-Popper Structure for Electrochemical Applications: Relationship between Ion (Oxygen-Ion, Proton) Conductivity, Water Uptake, and Structural Changes

**DOI:** 10.3390/ma15010114

**Published:** 2021-12-24

**Authors:** Nataliia Tarasova, Irina Animitsa

**Affiliations:** 1 The Institute of High Temperature Electrochemistry of the Ural Branch of the Russian Academy of Sciences, 620066 Ekaterinburg, Russia; irina.animitsa@urfu.ru; 2 Institute of Natural Sciences and Mathematics, Ural Federal University, 620066 Ekaterinburg, Russia

**Keywords:** BaLaInO_4_, layered perovskite, Ruddlesden-Popper structure, water uptake, oxygen-ion conductivity, protonic conductivity, the proton conducting solid oxide fuel cells

## Abstract

In this paper, the review of the new class of ionic conductors was made. For the last several years, the layered perovskites with Ruddlesden-Popper structure A^II^LnInO_4_ attracted attention from the point of view of possibility of the realization of ionic transport. The materials based on Ba(Sr)La(Nd)InO_4_ and the various doped compositions were investigated as oxygen-ion and proton conductors. It was found that doped and undoped layered perovskites BaNdInO_4_, SrLaInO_4_, and BaLaInO_4_ demonstrate mixed hole-ionic nature of conductivity in dry air. Acceptor and donor doping leads to a significant increase (up to ~1.5–2 orders of magnitude) of conductivity. One of the most conductive compositions BaNd_0.9_Ca_0.1_InO_3.95_ demonstrates the conductivity value of 5 × 10^−4^ S/cm at 500 °C under dry air. The proton conductivity is realized under humid air at low (<500 °C) temperatures. The highest values of proton conductivity are attributed to the compositions BaNd_0.9_Ca_0.1_InO_3.95_ and Ba_1.1_La0_.9_InO_3.95_ (7.6 × 10^−6^ and 3.2 × 10^−6^ S/cm correspondingly at the 350 °C under wet air). The proton concentration is not correlated with the concentration of oxygen defects in the structure and it increases with an increase in the unit cell volume. The highest proton conductivity (with 95−98% of proton transport below 400 °C) for the materials based on BaLaInO_4_ was demonstrated by the compositions with dopant content no more that 0.1 mol. The layered perovskites A^II^LnInO_4_ are novel and prospective class of functional materials which can be used in the different electrochemical devices in the near future.

## 1. Introduction

Recent economic and social challenges pose a priority task for scientists to create new high-efficiency and clean energy resources [1,2]. Hydrogen is a renewable and high-efficiency energy source, and its use has more advantages compared to fossil fuels [3,4]. The hydrogen-based economic requires development and improvement of many electrochemical devices including systems for hydrogen-producing (such as protonic ceramic electrolysis cells PCECs) and hydrogen-operated devices (solid oxide fuel cells SOFCs). Despite the active development of these devices, carried out during the past decades, the unresolved problems of their high cost and poor long-term stability still exist [5,6].

The development and complex investigation of oxygen- and proton-conduction ceramic materials is very relevant due to the possibility of using them as materials for PCECs and SOFCs [7,8,9,10,11]. The most investigated oxygen-ion and proton conductors are complex oxides with perovskite structure. However, further development of the materials science requires the study of compounds with a different type of structure, including the block-layered structures.

## 2. Structure of Layered Perovskite-Related Materials

### 2.1. Materials with K_2_NiF_4_-Type Structure

**Figure 1:**
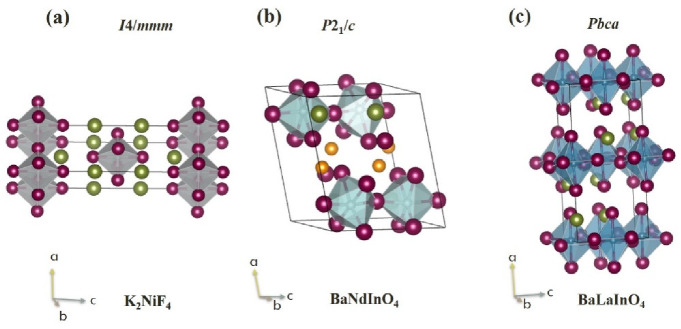
Structure of K_2_NiF_4_ (**a**), BaNdInO_4_ (**b**), and BaLaInO_4_ (**c**), where red spheres represent the oxygen atoms, green spheres represent the atoms of A-sublattice (K/Ba/La), and orange spheres represent the neodymium atoms.

The composition of K_2_NiF_4_ with monolayer block-layer structure was described for the first time by D. Balz and K. Plieth in the year 1955 [12]. Two years later, S.N. Ruddlesden and P. Popper showed that the structure of some new compounds (Sr_2_TiO_4_, Ca_2_MnO_4_, and SrLaAlO_4_) belongs to the structural type K_2_NiF_4_ [13]. This structure can be described as sequence of layers of distorted octahedra [NiF_6_] and layers [KF] with rock-salt framework (Figure 1a). The crystal structures presented in this work were depicted with VESTA [14]. In 1958 year, S.N. Ruddlesden and P. Popper showed the possibility of the existence of block-layer structures in which a perovskite block can contain not one, but several layers of octahedra [15]. Subsequently, such structures with the general formula A_n+1_B_n_X_3n+1_ were called as Ruddlesden-Popper (RP) structures [16]. In this general formula, A and B are cations, X is an anion (e.g., oxygen, fluorine), and *n* is the number of octahedral layers in the perovskite block.

In general, the K_2_NiF_4_-type structure has tetragonal symmetry *I*4/*mmm* and coordination formula $A_{\left(1\right)}^{\mathrm{IX}}A_{\left(2\right)}^{\mathrm{IX}}B^{\mathrm{VI}}O_{4}^{\mathrm{VI}}$. For obtaining this structure, the combination of structural characteristics is need: (i) The tolerance (Goldschmidt) factor *t* must lie between 0.95 and 0.985 [13]; (ii) cation size ratio ${\bar{R}}_{A}$/$R_{B}$ must be in the range 1.7−2.4 [17]. The monolayer RP-structure can be obtained by the different charge combinations of cations, such as $A_{2}^{+}B^{2+}F_{4}$, $A_{2}^{2+}B^{4+}O_{4}$, $A_{2}^{3+}B^{2+}O_{4}$, $A^{2+}A^{3+}B^{3+}O_{4}$,$\;A^{+}A^{3+}B^{4+}O_{4}$, $A^{+}B^{6+}O_{4}$, $A_{2}^{+}B^{2+}{\mathrm{Cl}}_{4}$, $A_{1.5}^{2+}A_{0.5}^{3+}B_{0.5}^{4+}B_{0.5}^{3+}O_{4}$ [18]. The different types of structures derived from the K_2_NiF_4_-type structure are described: (i) The T-structure (directly K_2_NiF_4_-structure) with tetragonal symmetry *I*4/*mmm* (e.g., La_2_NiO_4_); (ii) the tetragonal T′-structure which is characteristic for cuprates Ln_2_CuO_4_ (Ln = Pr, Nd, Sm, Eu, Gd); (iii) the orthorhombic O- and O′-structures (e.g., La_2_CoO_4_ and SmCoO_4_ correspondingly); (iv) the monoclinic M-structure (e.g., Pr_2_NiO_4_) [17]. It should be noted that the decrease in the symmetry of the structure leads to an increase in the tilting of octahedra [BO_6_]. It can be said that despite some differences is the crystal structures, the compositions derived from the K_2_NiF_4_-type structure can be named as PR-or PR-related materials.

**Figure 2:**
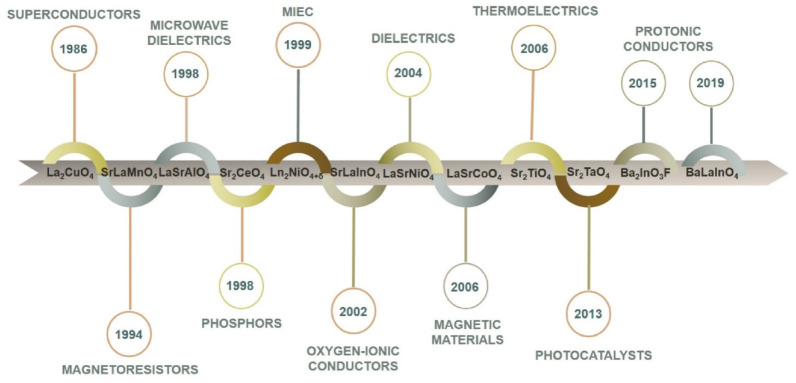
Historical overview on the investigation of the materials with Ruddlesden–Popper structure.

For the last 30 years, different compositions with K_2_NiF_4_ or related structures were described as superconductors [19,20,21,22,23,24,25,26], giant and colossal magnetoresistors [27,28,29,30], microwave dielectrics [31,32,33,34,35], phosphors [36,37,38,39], mixed ionic and electronic conductors (MIEC) [40,41,42,43,44,45,46,47,48,49,50], dielectrics [51,52,53,54,55], magnetic materials [56,57,58], thermoelectrics [59,60,61,62], photocatalysts for hydrogen production [63,64,65], oxygen-ionic conductors [66,67,68,69,70,71,72,73], protonic conductors [74,75,76,77,78,79,80,81,82,83,84,85] (Figure 2).

The possibility of existence of block-layer structures at the cation size ratio lower than 1.7 was described by Yu. Titov et al. [86]. It was shown that compositions with RP-structures with a different type of crystal lattice symmetry may exist up to ${\bar{R}}_{A}$/$R_{B}$ = 1.473. In other words, the significant difference between the radii of the cations in the A and B sublattices leads to the distortion of the structure and decreasing in the symmetry group. At the same time, the sequence of AO salt layers and ABO_3_ perovskite layers remain unchanged.

Some compositions obtained by Titov and characterized by ${\bar{R}}_{A}$/$R_{B}$ < 1.7 are represented in the Table 1. The general formula of these compositions can be written as A^II^Ln_n_In_n_O_3n+1_, where A is the alkali-earth element, Ln is lanthanide, and *n* = 1. A few years later, the use of neutron scattering allowed K. Fujii to prove the single phase of BaNdInO_4_ and to determine the monoclinic symmetry (s.g. *P*2_1_/*c*) for it [67]. Therefore, the new structural types of BaNdInO_4_ and BaLaInO_4_ were described. They are derived from K_2_NiF_4_-type structure and belonged to the monolayer PR-structures. Their unit cells are presented in Figure 1.

### 2.2. Materials with BaNdInO_4_-Type Structure

The structure of BaNdInO_4_ has seven independent sites Ba1, Nd1, In1, O1, O2, O3, and O4 (Figure 1b). In this type of the structure the layers of octahedra [InO_6_] do not alternate with salt layers BaO but with oxide layers (Ba,Nd)_2_O_3_. The significant distortion of BaNdInO_4_ structure from K_2_NiF_4_ structure leads to the changes in coordination environment of ions. The coordination formula for BaNdInO_4_ can be written as $A_{\left(1\right)}^{\mathrm{XI}}A_{\left(2\right)}^{\mathrm{VII}}B^{\mathrm{VI}}O_{4}^{\mathrm{VI}}$. It was shown [73] that compositions BaYInO_4_, BaSmInO_4_, BaHoInO_4_, BaErInO_4_, BaYbInO_4_ also belonged to the BaNdInO_4_-type structure. The increase in the ionic radii of alkali-earth element led to the increase in the lattice parameters *a*, *b* and unit cell volume and decrease in the parameter *c* и β angle.

The layered structure of BaNdInO_4_ exhibits the ability to accommodate various types of substitutions. The possibility of acceptor doping of Nd-sublattice [67,72,73] and donor doping of In-sublattice [71] were described. The introduction of cations of alkali-earth metals Ca^2+^, Sr^2+^, Ba^2+^ in the Nd^3+^-positions led to the formation of oxygen vacancies. For the general formula A^II^LnInO_4_ the quasi-chemical equation can be written as:

(1) $$2\mathrm{AO}\overset{{\mathrm{Ln}}_{2}O_{3}}{\to }2A_{\mathrm{Ln}}^{\prime }+2O_{o}^{\times }+V_{o}^{••}$$

At the same time, doping by ions with close ionic radii (Ca^2+^) led to the contraction of cell volume. Contrarily, the doping by bigger ions (Sr^2+^, Ba^2+^) led to the expansion of cell volume ($r_{{\mathrm{Ca}}^{\left(\mathrm{VII}\right)2+}}$ = 1.06 Å, $r_{{\mathrm{Sr}}^{\left(\mathrm{VII}\right)2+}}$ = 1.21 Å, $r_{{\mathrm{Ba}}^{\left(\mathrm{VII}\right)2+}}$ = 1.38 Å, $r_{{\mathrm{Nd}}^{\left(\mathrm{VII}\right)3+}}$ = 1.046 Å [87]). The unit-cell volume of the solid solutions BaNd_1−*x*_Sr*_x_*InO_4−*x*/2_ (0 ≤ x ≤ 0.3) [67] and Ba_1+*x*_Nd_1−*x*_InO_4−*x*/2_ (0 ≤ x ≤ 0.1) [72] linearly increased with the increase of dopant concentration. The cell volume for the solid solution BaNd_1−*x*_Ca*_x_*InO_4−*x*/2_ (0 ≤ x ≤ 0.2) slightly decreased [82].

The donor doping of In-sublattice by such ions as Zr^4+^, Ti^4+^, Nb^5+^, Ta^5+^ with dopant concentration *x* = 0.1 led to the formation of single-phase compositions with BaNdInO_4_-type structure [71]. The donor doping of A^II^LnInO_4_ by M^4+^ and M^5+^ cations of In^3+^-sublattice assumes the formation of interstitial oxygen in the structure:

(2) $$2{\mathrm{MO}}_{2}\overset{{\mathrm{In}}_{2}O_{3}}{\to }2M_{\mathrm{In}}^{•}+3O_{o}^{\times }+O_{i}^{\prime \prime }$$

(3) $$M_{2}O_{5}\overset{{\mathrm{In}}_{2}O_{3}}{\to }2M_{\mathrm{In}}^{••}+3O_{o}^{\times }+2O_{i}^{\prime \prime }$$

Nevertheless, the details of crystal structure of donor-doped samples BaNdIn_0.9_M_0.1_O_4+δ_ were missed. Moreover, the influence of doping on the local structure including the change in the coordination environment of ions due to the formation of point defects (oxygen vacancy, oxygen interstitial) and in the deformation of polyhedra have not been investigated.

### 2.3. Materials with BaLaInO_4_-Type Structure

The structure of BaLaInO_4_ has four independent sites Ba1/La1, In1, O1, and O2 (Figure 1c) and belongs to the orthorhombic symmetry (s.g. *Pbca*). The coordination formula is the same as for K_2_NiF_4_-type structure. The compositions SrLaInO_4_ and SrPrInO_4_ have the same type of the structure (s.g. *Pbca*) [86]. The changes in the ionic radii of alkali-earth (A_(1)_) and lanthanide metals (A_(2)_) leads to some changes in the structure of these compositions (Table 2). As can be seen, the decrease in the ratio ${\bar{R}}_{A}$/$R_{B}$ is accompanied by the decrease in the parameter *a* and in the interlayer space (bond length A_(1),_A_(2)_−O2) and by the increase in the deformation of the [(A_(1)_,A_(2)_)O_9_] polyhedra. This occurs at decreasing of the ionic radii of A_(1)_ and A_(2)_ metals. The deformation Δ of polyhedra was calculated as [86]:

(4) Δ=1n∑[(li−l¯)/l]2 

where $l_{i}$ is the bond length M−O, $\bar{l}$ is the average bond length M−O, *n* is the coordination number. 

The possibility of acceptor and donor doping for SrLaInO_4_ and BaLaInO_4_ compositions were described. The insertion of Sr^2+^-ions in the La^3+^-sublattice of SrLaInO_4_ (acceptor doping) led to the formation of Sr_1+*x*_La_1−*x*_InO_4−0.5*x*_ (*x* = 0.1, 0.2) [66]. The compositions BaLa_0.9_M_0.1_InO_3.95_ (M = Ca^2+^, Sr^2+^, Ba^2+^) [76] and solid solution Ba_1+*x*_La_1−*x*_InO_4−0.5*x*_ (0 ≤ *x* ≤ 0.15) [79] were produced by the doping by M^2+^-ions of BaLaInO_4_. The insertion of La^3+^-ions in the Sr^2+^-sublattice [69] and M′^4+^-ions (M′ = Ti, Zr) in the In^3+^-sublattice [70] (donor doping) led to the formation of Sr_1−*x*_La_1+*x*_InO_4+0.5*x*_ and SrLaIn_1−*x*_M*_x_*O_4+0.5*x*_ (*x* = 0.1, 0.2). The compositions BaLaIn_0.9_M′_0.1_O_4+δ_ (M′ = Ti, Zr, Nb) [78] and solid solutions BaLaIn_1−*x*_Ti*_x_*O_4+0.5*x*_ (0 ≤ *x* ≤ 0.15) [80] and BaLaIn_1−*x*_Nb*_x_*O_4+*x*_ (0 ≤ *x* ≤ 0.10) [83] were obtained by the doping of BaLaInO_4_.

Both acceptor and donor doping led to the formation of oxygen defects in the structure (oxygen vacancy and oxygen interstitial correspondingly). Consequently, comparative analysis of the changes in the local structure of doped samples is needed.

The investigation of local structure of acceptor- and donor-doped samples based on BaLaInO_4_ using Raman spectroscopy showed the presence of local disordering of the crystal lattice [88,89]. The acceptor doping led to the decrease in the coordination number of metal (barium and lanthanum) because of the formation of oxygen vacancies during doping (Equation (1)). Consequently, the bond length Ba,La−O2 in the vacancy-containing polyhedra decreased. At the same time, the introduction of ions with bigger ionic radii Sr^2+^, Ba^2+^ ($r_{{\mathrm{Sr}}^{\left(\mathrm{IX}\right)2+}}$ = 1.31 Å, $r_{{\mathrm{Ba}}^{\left(\mathrm{IX}\right)2+}}$ = 1.47 Å, $r_{{\mathrm{La}}^{\left(\mathrm{IX}\right)3+}}$ = 1.216 Å [87]) in the La^3+^-sublattice provides the increase of Ba,La−O1 bond length. The average bond length Ba,La−O increases what caused the expansion of unit cell in the *ab* direction and the decrease of octahedra [InO_6_] titling [88,89]. This allows to say that acceptor doping of BaLaInO_4_ led to the formation of less distorted structure. The distortion decreased with increasing the ionic radius of dopant and dopant concentration. 

The donor doping led to the appearance of “additional” (interstitial) oxygen O3 (Equations (2) and (3)). Consequently, the coordination number of some Ba^2+^- and La^3+^-cations increased, and the bond lengths Ba,La−O1 and Ba,La−O2 and lattice parameter *a* increased also [88,89]. The increase of the charge of dopant (Ti^4+^, Nb^5+^) and the concentration of dopant led to a decrease of distortion of the structure. It is accompanied by the decrease of the tilting of In-containing polyhedra.

Therefore, acceptor doping of La^3+^-sublattice of BaLaInO_4_ by ions with bigger ionic radii (Sr^2+^, Ba^2+^) and donor doping of In^3+^-sublattice by Ti^4+^-, Nb^5+^-ions, accompanied by the formation of interstitial oxygen, led to the increase of the average bond length Ba,La−O and expansion of unit cell in the *ab* direction. In other words, the structure of doped samples became less disordered and less compressed. These factors can provide the positive role in the hydration processes and transport properties.

## 3. Water Intercalation into Structure of Layered Perovskites

The materials with layered RP-structure have two principal different ways for water intercalation. First, the formation of hydrates occurs. In this case, the water molecules are embedded into the sites within the rock-salt layers without dissociation into H^+^- and OH^−^- ions. The crystal structure hydrated such as Ba_2_ZrO_4_∙*n*H_2_O that contains ordered PR-layers separated by (H_2_O)*_n_* layers with statistical distribution within the layer [90]. The thickness of water layers is different and dependent on hydration conditions such as the temperature and water partial pressure. 

The same situation was observed for titanates NaEuTiO_4_ [91] and ALaTiO_4_ (A = Li, Na, K) [92]. However, for compositions ALnTiO_4_ where A is the alkali metal and Ln is the rare-earth metal, both types of water intercalation processes are possible [93,94,95,96]. The dissociative intercalation of water is obtained by the protonation and leads to the formation of the compositions with the general formula HLnTiO_4_. The most common situation is the existence of partially or completely protonated compositions with some amount of water H*_x_*Ln_1−*x*_TiO_4_∙*n*H_2_O. In this case, the hydrogen atoms are present in crystallographic position of alkali metal into A-sublattice and as part of water molecules in the interlayer space at the same time. It should be noted that for all cases the hydration led to the expansion of interlayer space and was accompanied by the structural rearrangement. 

Second opportunity for the water intercalation in the PR-phases is the dissociative dissolution of water into crystal lattice. This type of hydration was observed for the perovskite or perovskite-related materials with oxygen vacancies in the structure. The amount of water uptake depends on the amount of oxygen vacancies which can be introduced into the crystal lattice by the acceptor doping [97] or to be own structural defects [98,99,100]:

(5) $$V_{o}^{••}+H_{2}O+O_{o}^{\times }\Leftrightarrow 2{\left(\mathrm{OH}\right)}_{o}^{•}$$

(6) $$V_{o}^{\times }+H_{2}O+2O_{o}^{\times }\Leftrightarrow 2{\left(\mathrm{OH}\right)}_{o}^{•}+O_{i}^{\prime \prime }$$

where $V_{o}^{••}$ is the oxygen vacancy, $O_{o}^{\times }\;$is the oxygen atom in the regular position,$\;{\left(\mathrm{OH}\right)}_{o}^{•}$ is the hydroxyl group in the oxygen sublattice, $O_{i}^{\prime \prime }$ is the oxygen atom in the interstitial position. 

**Figure 3:**
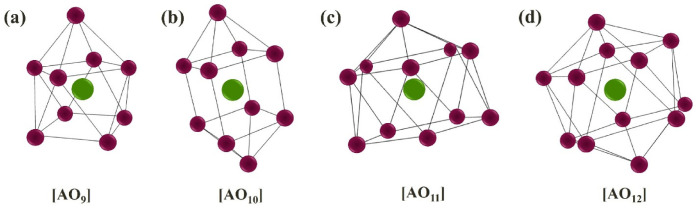
Representation of oxygen polyhedra (red spheres) with different coordination number of central atom (green sphere).

Because the RP-materials A_(1)_A_(2)_BO_4_ do not contain the oxygen vacancies, the dissociative intercalation of water is realized by the incorporation of hydroxyl groups into salt blocks [(A_(1)_,A_(2)_)O]:

(7) $$H_{2}O+O_{O}^{x}\Leftrightarrow {(\mathrm{OH})}_{O}^{•}+{\left(\mathrm{OH}\;\right)}_{i}^{\prime }$$

where ${(\mathrm{OH})}_{O}^{•}$ is the hydroxyl group in the regular oxygen position, ${\left(\mathrm{OH}\;\right)}_{i}^{\prime }$ is the hydroxyl group located in the interlayer space. This process is accompanied by the increase of the coordination number of metals in the A-sublattice from 9 (Figure 3a) up to 12 (Figure 3b–d). Therefore, the possibility of water uptake in the RP-materials is provided at the realization of next conditions:

1. The possibility of increasing coordination number of the metals in the A-sublattice;
2. The sufficient size of interlayer space for the localization of hydroxyl groups.

The data about water uptake of the materials based on BaNdInO_4_ and SrLaInO_4_ are not numerous. It was proved by the thermogravimetric measurements, that acceptor-doped solid solution BaNd_1−*x*_Ca*_x_*InO_4−*x*/2_ (0 ≤ *x* ≤ 0.25) was capable for the dissociative incorporation of H_2_O up to 1.1 mol per formula unit [82]. The refinement of neutron powder diffraction data obtained for donor-doped complex oxides Ba*_x_*Sr_0.8−*x*_La_1.2_InO_4+δ_ (*x* = 0.2, 0.3) was shown the presence of about 0.50 water molecules per formula unit in the structure [77]. These data confirm that RP-materials can incorporate a significant water content. However, the relationships of the changes of the crystal structure during hydration, the amount of water uptake, and the nature of oxygen-hydrogen groups are described in detail only for doped compositions based on BaLaInO_4_. 

The change in the crystal symmetry from s.g. *Pbca* for anhydrous forms of the samples to s.g. *P*2/*m* for hydrated forms was observed for all compositions obtained by the doping of BaLaInO_4_ [76,78,79,80,81,83,84]. As it was mentioned earlier, the acceptor and donor doping led to the formation of oxygen vacancies and oxygen interstitial in the structure correspondingly. Consequently, the influence of the presence of oxygen defects and their concentration on the water uptake should be taken into account for the acceptor- and donor-doped compositions.

**Figure 4:**
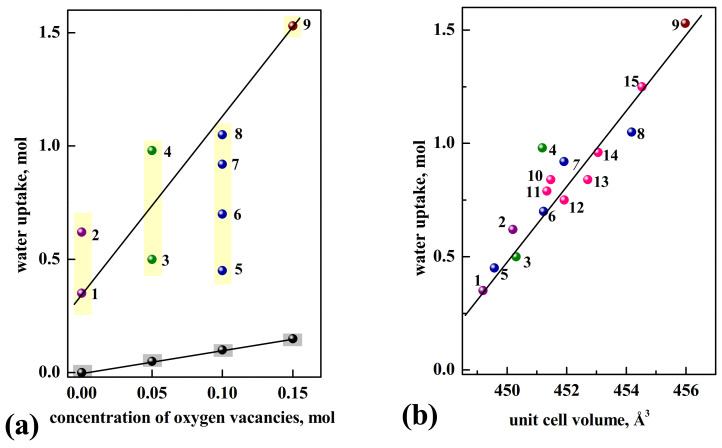
The dependencies of water uptake vs. concentration of oxygen vacancies (**a**) and unit cell volume (**b**) for the compositions BaLaIn_0.9_Sc_0.1_O_4_ (1) [83], BaLaInO_4_ (2) [74], Ba_1.05_La_0.95_InO_3.975_ (3) [79], Ba_1.1_La_0.9_In_0.95_Ti_0.05_O_3.98_ (4) [81], Ba_1.1_La_0.9_In_0.95_Ti_0.1_O_4_ (5) [81], BaLa_0.9_Ca_0.1_InO_3.95_ (6) [74], BaLa_0.9_Sr_0.1_InO_3.95_ (7) [74], Ba_1.1_La_0.9_InO_3.95_ (8) [79], Ba_1.15_La_0.85_InO_3.925_ (9) [79], BaLaIn_0.95_Ti_0.05_O_4.025_ (10) [80], BaLaIn_0.95_Nb_0.05_O_4.05_ (11) [84], BaLaIn_0.9_Zr_0.1_O_4.05_ (12) [78], BaLaIn_0.9_Nb_0.1_O_4.10_ (13) [84], BaLaIn_0.9_Ti_0.1_O_4.05_ (14) [80], BaLaIn_0.85_Ti_0.15_O_4.075_ (15) [80]. The violet, green/blue/red, and rose symbols correspond to the compositions without oxygen defects, with oxygen vacancies and oxygen interstitials in the structure correspondingly.

Figure 4a represents the dependency of water uptake vs. concentration of oxygen vacancies for acceptor-doped samples. All data were obtained by thermogravimetric (TG) measurements [101]. Based on the amount of oxygen vacancies, we can predict the amount of water uptake in the structure according to Equation (5) (black symbols in the Figure 4a). The real values of water uptake are represented by colored symbols. As seen, the general trend of increase in the amount of water uptake with increase in the vacancy concentration is retained. However, the experimental data are higher than the predicted values up to one order of magnitude, and the range of values obtained for the compositions with the same vacancy concentration is 0.3−0.6 mol H_2_O (yellow color in the Figure 4a). Therefore, the correlation of amount of water uptake with the concentration of oxygen vacancies is not obvious. 

Figure 4b represents the dependency of water uptake vs. unit cell volume for the compositions based on BaLaInO_4_. As we can see, for all compositions the general trend of increase of water uptake amount with increase of unit cell volume is observed. The data for donor-doped samples are presented in Figure 4 also, i.e., the data for the samples with oxygen interstitial in the crystal lattice. The presence of such oxygen defects in the structure leads to the increase in coordination number of the part of Ba,La-contained polyhedra from 9 (Figure 3a) to probably 10 (Figure 3b) in the anhydrous state. Consequently, it can be expected the less amount of water uptake for the donor-doped samples compared with the amount for acceptor-doped samples at the same unit cell volume. For example, the amount of water uptake for BaLaIn_0.9_Zr_0.1_O_4.05_ was less than for BaLa_0.9_Sr_0.1_InO_3.95_ by ~0.2 mol per formula unit.

The state of oxygen-hydrogen groups in the structure of hydrated compositions based on BaLaInO_4_ was detected by the infrared spectroscopy (IR) method. According to IR-data, only hydroxyl groups were present. No water molecules or hydroxonium ions were detected [101]. Thus, it can be said that for the layered perovskites based on BaLaInO_4_ the mechanism of dissociative dissolution of water into crystal lattice is realized.

**Figure 5:**
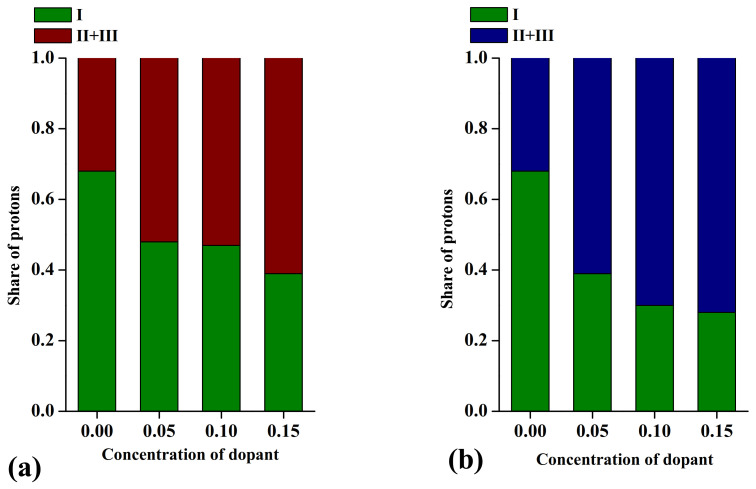
The dependencies of share hydroxyl groups involved into different hydrogen bonds vs. dopant concentration for the solid solutions Ba_1+*x*_La_1−*x*_InO_4−0.5*x*_ (**a**) [79] and BaLaIn_1−*x*_Ti*_x_*O_4+0.5*x*_ (**b**) [80], where “I” and “II” are attributes to the share of hydroxyl groups involved in the strong and weaker hydrogen bonds, “III” is corresponded to the share of isolated hydroxyl groups.

The comparative analysis of TG- and IR-data of solid solutions Ba_1+*x*_La_1−*x*_InO_4−0.5*x*_ (0 ≤ *x* ≤ 0.15) and BaLaIn_1−*x*_Ti*_x_*O_4+0.5*x*_ (0 ≤ *x* ≤ 0.15) made it possible to identify the energy non-equivalent hydroxyl groups. As we can see from Equation (7), the hydroxyl groups have different positions in the crystal lattice. The hydroxyl groups on the regular oxygen position and the hydroxyl groups located within the rock-salt layers can be distinguished. Obviously, the hydroxyl groups located into different crystallographic positions must be involved in hydrogen bonds with different strength. Figure 5. represents the dependency of share hydroxyl groups involved in different hydrogen bonds vs. dopant concentration for Ba-doped (Figure 5a) and Ti-doped (Figure 5b) solid solutions. As it was discussed earlier, the increase in the dopant concentration lead to the expansion of unit cell in the *ab* direction. This expansion must lead to the increase in the distance between hydroxyl group and the oxygen atom, involved in the hydrogen bond. Consequently, the decrease in the share of hydroxyl groups involved in the strong hydrogen bonds (I) compared with share of hydroxyl groups involved in the weaker (II) and isolated (III) hydrogen bonds can be predicted. The experimental obtained data (Figure 5) confirm this assumption. It should be noted that the nature of oxygen defect (oxygen vacancy, oxygen interstitial) does not significantly affect the proportion of shares from different hydroxyl groups. 

Therefore, the layered perovskites are capable for the intercalation of water even if there are no oxygen vacancies in the structure. The hydration of the complex oxides based on BaLaInO_4_ is realized by the dissociative dissolution of water molecules and the localization of hydroxyl groups into interlayer space. This process is accompanied by the increase in the coordination number of Ba and La atoms. The water uptake increases with the increase in the unit cell volume and it is not determined by concentration of oxygen defects in the structure. The water uptake for doped compositions based on BaLaInO_4_ is up to 1.5 mol H_2_O per formula unit, which is much bigger than for known perovskite-related materials [97]. This makes it possible to consider RP-materials as promising proton conductors.

## 4. Oxygen-Ionic Conductivity in the Layered Perovskite-Related Materials

### 4.1. General Remark

Despite the layered perovskites first reported in the mid 1950s [12,13,15], the importance of these materials was initially limited by the discovery of superconductivity in cuprate La_2-x_Ba_x_CuO_4_, discovered in 1986 [19]. For a long time, the electrical properties of this class of materials did not attract attention. X. Turrillas et al. studied the conductivity of Sr_3_Ti_1.9_M_0.1_O_7-δ_, (M = Al, Mg) and low level of conductivity was observed [102].

Among the early works on the study of oxygen-ionic conductivity in RP phases, the paper [103] should be mentioned, in which the oxygen deficient barium indates Ba_8_In_6_O_17_ was investigated. Its structure consists of an intergrowth of rock-salt planes of BaO with triple layers of the oxygen-deficient perovskite-like BaInO_2.5_. This phase exhibited the high oxygen-ionic conductivity comparable to Y_2_O_3_-stabilized zirconia (1.1 × l0^−4^ S∙cm^−1^ at 450 °C).

The conductivity of the family of the materials Sr_n+l_Zr_n_O_3n+1_ was investigated by F.W. Poulsen et al. in 1992 [104] and the composition Sr_2_ZrO_4_ had a conductivity of 7.5 × 10^−5^ and 5.9 × 10^−4^ S/cm at 750 and 1000 °C, respectively. The authors emphasized that the nature of ionic conductivity is not known; it can be either oxygen-ion or proton transport. 

In 1997 the investigation of the Ruddlesden-Popper phases was started by C. Navas and H.-C. zur Loye [105] in order to find a new oxygen-deficient layered intergrowth structure, analogous to the Aurivillius phases, but without Bi. The phases Sr_3_M_2_O_7_ (M = Ti, Zr) doped with Al^3+^, Ga^3+^, and In^3+^ were investigated. Ionic conductivity predominated only in intermediate *p*O_2_ ranges (10^−5^−10^−15^ atm), and it is only slightly higher than 10^−5^ S/cm at 800 °C. Like Turrillas X. et al. in early work [102], these authors concluded that these phases are not good candidates for using as electrolyte systems. This is a serious hindrance to the application of layered perovskites to SOFC.

Because the conductivity of these doped materials was fairly low, the main doping strategy of many research was to increase electronic conductivity. Later, with the development of materials science research, the systems with high oxygen-ion conductivities were discovered. For example, S. Kato et al. in 2002 [66] described the solid solution La_1−*x*_Sr_1+*x*_InO_4-δ_ that exhibited a conductivity of 10^−3^ S/cm at 600 °C. Perhaps this was the first work in which the promising nature of the usage of phases with RP- structure as oxygen-ion conductors and the possibility of a significant change in the conductivity with suitable doping have been proved. This article became the starting point for motivation for the development of a broad materials science search of new phases with a layered structure. Further, systemic studies of phases with the RP-structure made it possible to establish the main relationships of ion transport for this class of layered oxides.

In this review, we will focus on a discussion of phases with ionic conduction based on In-containing compounds R^3+^(Sr,Ba)InO_4_ with RP-type structure.

### 4.2. Mechanisms of Oxygen Ion Migration in RP-Phases

Since RP-phases, containing transition metals with a variable oxidation state (nickelates and cuprates mainly), have been widely studied as mixed conductors and the understanding of mechanism of oxygen-ion transport is important for such systems, therefore, works devoted to this problem have been widely discussed in the literature. Recent reviews describe mechanisms of oxygen ion migration in sufficient detail [106,107,108]. The defect processes in RP oxides can be described by anion Frenkel disorder:

(8) $$O_{O}^{x}\Leftrightarrow V_{o}^{••}+O_{i}^{\prime \prime }$$

Due to the structural features of RP oxides and flexibility in oxygen stoichiometry (hypostoichiometry or hyperstoichiometry) oxygen ion migration in RP-structures can occur by oxygen vacancy or oxygen interstitial mechanisms. It is usually believed that in oxygen-deficient phases oxygen diffusion is carried out by the migration of oxygen vacancies within the perovskite layer, and in oxygen-excess phases an interstitial oxygen migration is dominant. 

In general, there are three oxygen diffusion mechanisms: the vacancy mechanism, the direct interstitial mechanism, and interstitialcy mechanism [107].

(i) The direct interstitial mechanism is associated with the migration of interstitial ions directly to the adjacent interstitial site.
(ii) Interstitialcy mechanism includes such process: interstitial oxygen kicks the apical lattice oxygen atom out from the LaO-plane, placing it to the next nearest available interstitial site, while itself moving to site of the displaced apical oxygen on the LaO plane (push–pull mechanism). The facile transport of the interstitial oxygens is enabled by the cooperative titling of the BO_6_ octahedron. DFT calculations indicate that this process requires a lower activation energy than that of the direct interstitial mechanism [109].
(iii) The vacancy mechanism of diffusion is due to oxygen jumping to a neighboring vacancy.

**Figure 6:**
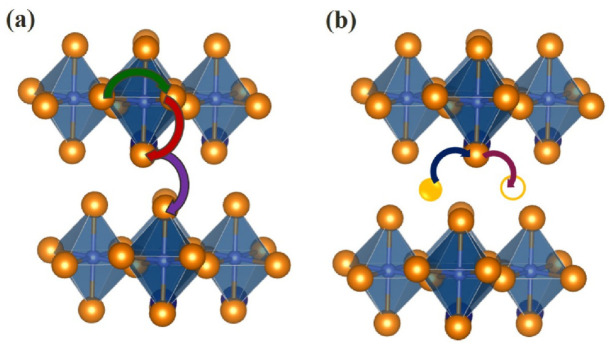
3D representation of the vacancy (**a**) and interstitial (**b**) oxide ion migration in doped A^II^LnInO_4_.

All these mechanisms are described for RP-phases [110]. Researchers are more focused on describing the interstitial mechanisms of oxygen migration, however, there is work that pays attention to vacancy migration [111]. The oxygen migration mechanisms, described by C. Tealdi at al. [111], are shown in Figure 6. The authors showed that *vacancy* migration is not necessarily restricted to the perovskite layer, since jumps occur between apical oxygens of adjacent layers. Figure 6 shows the following migration paths:

(a) Oxygen vacancy migration between
  1. Equatorial-apical positions,
  2. Between equatorial positions,
  3. Between apical positions belonging to separate layers;
- Oxygen interstitial migration—”wave-like” mechanism (2D path between apical and interstitial sites within the *ab* plane).

The activation energy for oxygen vacancy migration by hopping between two adjacent equatorial positions within the perovskite layer is lowest migration energy (0.97 eV) [111]; the activation energies for oxygen vacancy migration between equatorial–apical positions and apical–apical positions in two separate perovskite layers are slightly higher (1.14 and 1.26 eV); and the activation energy for oxygen vacancy migration between apical–apical positions within the perovskite layer is 2.11 eV. The oxygen interstitial migration occurs with a lower activation energy of 0.71 eV [111].

Among the features of oxygen diffusion in RP-phases, the following can be noted:

− There is a high degree of anisotropy in the oxygen transport, interstitial diffusion in the rock-salt *ab* plane is at least an order of magnitude faster than along the *c-*direction;
− Unusual feature of rp-materials is the existence of interstitials in both oxide and peroxide states, and both can take part in diffusion [112].

Oxygen migration in RP-phases, containing elements with higher stable oxidation states is less studied, although it can be assumed that in general the mechanisms will be similar to those described for mixed conductors. Ca-doped NdBaInO_4_ phase was described by X. Yang et al. [72] and both the static lattice and molecular dynamic simulations indicated oxygen vacancy migration within the perovskite layer. Molecular dynamic simulations specified two major vacancy migration ways, via one intraslab path along the *b-*axis and one interslab path along the *c-*axis. The intraslab and interslab migration involves the terminal oxygen sites within the perovskite layers and has comparable contributions. This result is consistent with the 2D oxygen diffusion in [NdO] layer suggested by K.Fujii et al. in accordance with the DBVS results [67].

### 4.3. SrLaInO_4_-Based Materials

**Figure 7:**
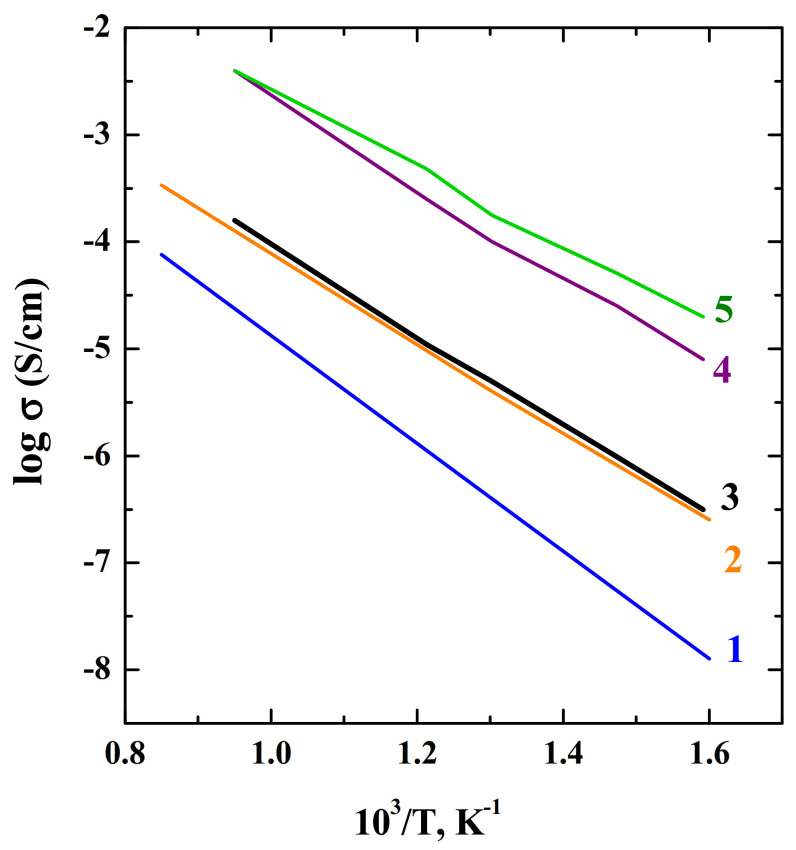
The temperature dependencies of electrical conductivities of the compositions SrLaInO_4_ (1) [70]; SrLaIn_0.8_Zr_0.2_O_4+d_ (2) [70]; Sr_1+*x*_La_1−x_InO_4−d_, *x* = 0 (3), *x* = 0.1 (4), *x* = 0.2 (5) [66].

The solid solution La_1−*x*_Sr_1+*x*_InO_4-δ_ 0 ≤ *x* ≤ 0.2, was described by S. Kato et al. in 2002 [66]. These compounds exhibited dominant oxide ion conduction at *p*O_2_ below 10^−5^ atm. In air, the phases showed contribution of hole conduction. The authors showed that introduction of oxygen vacancy by increasing of Sr^2+^/La^3+^ ratio on A-site of the layered perovskite LaSrInO_4_ was effective for increasing oxide ion conductivity. Doping increased the conductivity by almost 2 orders of magnitude compared to the undoped composition LaSrInO_4_ (Figure 7). The value of activation energy *E_a_* of the oxide-ion conduction was not large and reached 0.87 eV. The authors pointed out that the ion conduction in Sr-doped layered perovskite LaSrInO_4_ is comparable to the ion conduction in Sr-doped simple perovskite LaInO_3_ (logσ = −2.3 at 800 °C [113]), making layered perovskite-type compounds promising candidate for SOFC. Further works appeared on the introduction of various dopants into the phase LaSrInO_4_.

Introduction of Ga^3+^ in In^3+^-sublattice of layered perovskite LaSrInO_4_ or solid solution La_1-x_Sr_1+x_InO_4-δ_ was accompanied by a decrease in conductivity [114], which is explained by a decrease in the unit cell volume. Thus, for layered structures, in addition to defectiveness (concentrations of defects), an important parameter affecting oxygen-ion conductivity is an increase in the lattice volume. In this regard, Ba^2+^-substituted phases or Ba-analogs of layered perovskites may be of interest. 

An important feature of layered perovskites is the ability to adapt interstitial oxygen in a wide range; therefore, donor doping of the phase LaSrInO_4_ was also used. The interstitial oxygens are located in the NaCl-layer and coordinated of A-cations. The interstitial oxygens are mobile, because of their coordination numbers are lower than those of other oxygen atoms. For example, In^3+^ can be replaced by Zr^4+^ or Ti^4+^ and introduction of some oxygen excess can be realized [69,70]. The solid solutions LaSrIn_1−*x*_B*_x_*O_4+δ_ (B = Zr, Ti) were synthesized via a nitrate–citrate route and neutron diffraction analysis proved that the interstitial O3 atoms occupy the (La,Sr)O rock-salt layer and promote the expansion of the *ab* plane. Although the cell volume decreased upon doping (due to the contraction along *c*), the conductivity of doped samples increased, for example, by an order of magnitude for the composition LaSrIn_0.8_Zr_0.2_O_4+0.08_ [70] (Figure 7). Thus, the expansion of the salt block is an important factor for increasing the oxygen-ion conductivity in layered perovskites. 

### 4.4. BaNdInO_4_-Based Materials

In 2014 a new structure family of oxide-ion conducting material, based on the composition NdBaInO_4_ was discovered [67]. From the point of view of the design of new materials among the composition AAʹBO_4_, where A and Aʹ are larger cations and B is a smaller cation, the choice of Nd, Ba, In cations was based on the following considerations: (i) the different sizes of Nd and Ba- cations result in the ordering of Ba/Nd cations and (ii) the BaInO_2.5_ perovskite unit can form in view of the sizes of Ba and In cations. The structure of NdBaInO_4_ is slightly different from K_2_NiF_4_, which was discussed in Section 2 above. 

#### 4.4.1. Effect of Substitutions on the A-Sites

**Figure 8:**
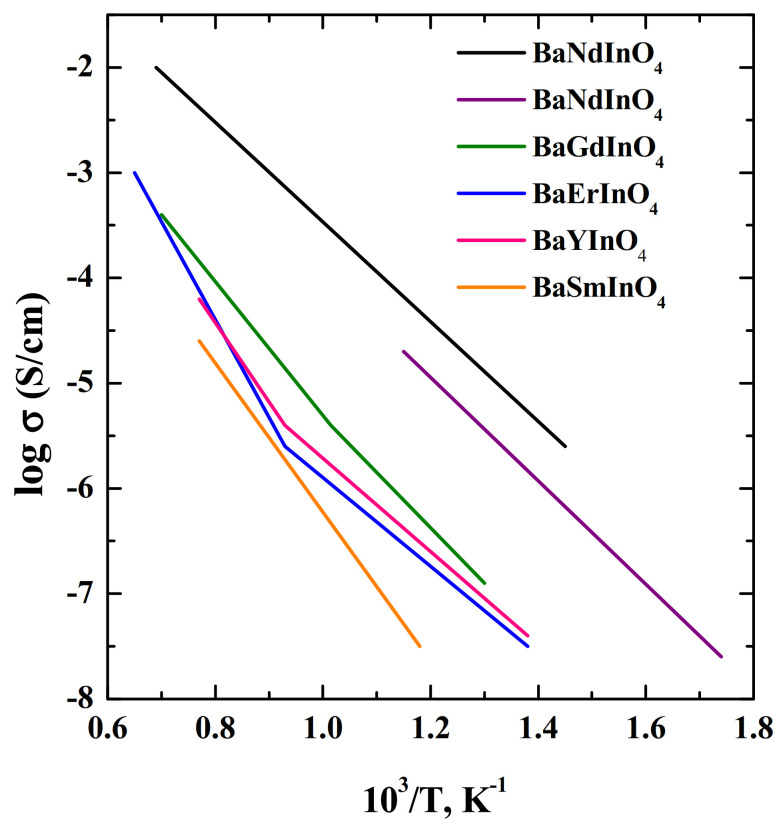
The temperature dependencies of electrical conductivities of compositions BaNdInO_4_ (violet) [72], BaNdInO_4_ (black) [73], BaGdInO_4_ (green) [73], BaErInO_4_ (blue) [73], BaYInO_4_ (pink) [73], BaSmInO_4_ (orange) [73].

A systemic research was carried out by researchers [67,68,72,73] to study the structure, ion transport mechanisms, and electrical properties of phases based on NdBaInO_4_. Among all of the BaRInO_4_-based materials (R = Y, Nd, Sm, Gd, Ho, Er, Yb) with layered perovskite structures the Nd-containing phases showed the highest oxide ion conductivities (Figure 8). Therefore, doped NdBaInO_4_ was the most widely studied. 

**Figure 9:**
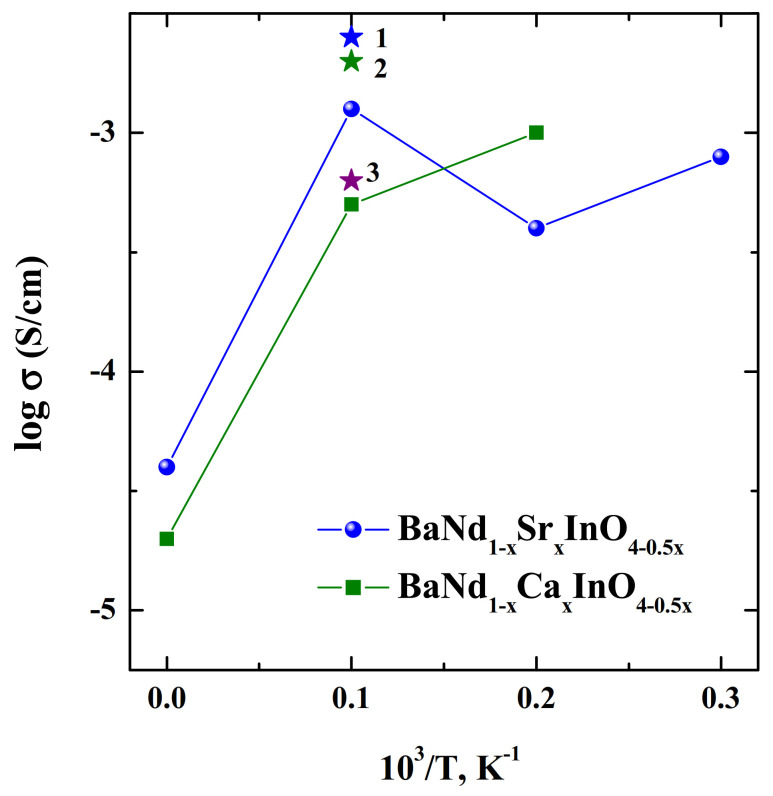
The concentration dependencies of electrical conductivities for the solid solutions BaNd_1−*x*_Sr*_x_*InO_4−0.5*x*_ (green) [68] and BaNd_1−*x*_Ca*_x_*InO_4−0.5*x*_ (blue) [82], and for the compositions BaNd_0.9_Ca_0.1_InO_3.95_ (1) [72], BaNd_0.9_Sr_0.1_InO_3.95_ (2) [72], BaNd_0.9_Ba_0.1_InO_3.95_ (3) [72] at 600 °C.

The undoped BaNdInO_4_ exhibited mixed oxide-ion and hole conduction; oxide-ion conduction was dominant in the intermediate *p*O_2_ region (e.g., *p*O_2_ = 10^−22^−10^−9^ atm at 858 °C). The improvement of oxide-ion conductivity of BaNdInO_4_ was performed by various cation doping. The oxygen-deficient Ca, Sr, Ba- doped on the Nd-sites phases were obtained and the existence of oxygen vacancies in the crystal structures was experimentally confirmed by neutron powder diffraction data [69,72]. For the doped samples and for the same concentration of oxygen vacancies, the bulk conductivities in air increases in the sequence σ(Nd_0.9_Ba_1.1_InO_3.95_)—σ(Nd_0.9_Sr_0.1_BaInO_3.95_)—σ(Nd_0.9_Ca_0.1_BaInO_3.95_) (Figure 9). In the same sequence, the activation energy for oxide-ion conduction decreased 0.86 [68,82]—0.795−0.73 eV, these values were lower than that of undoped BaNdInO_4_ 0.95 eV [72]. 

The authors [72] analyzed various reasons explaining the effect of the dopants Ca^2+^, Sr^2+^, and Ba^2+^ on oxygen-ion conductivity in NdBaInO_4_.

− Energetic of defect formation. The calculated solution energies of Ca^2+^ (0.76 eV), Sr^2+^ (0.84 eV), and Ba^2+^ (1.6 eV) on Nd^3+^ sites showed that Ba^2+^ was the most energy-unfavorable dopant [72].The authors of [72] indicated that the replacement of Nd^3+^ by the cations with the comparable size may reduce the local structural relaxation and this made it possible to explain the increase in the oxygen conductivity in the order Ba^2+^, Sr^2+^, Ca^2+^-dopants.
− Binding energy of the dopant-vacancy cluster. It is well-known that minimal binding energy for the dopant-vacancy cluster promotes the O^2−^-conductivity. At the same time, the calculated binding energies for Ca-, Sr-, and Ba-doped NdBaInO_4_ were comparable ca. −0.9 eV, so, the trapping of the oxygen vacancies is not the main factor in understanding the change in conductivity upon doping.
− Oxygen migration energy. The oxygen vacancy migration is two-dimensional within the perovskite-laere boundary region for the acceptor-doped NdBaInO_4_. Molecular dynamic simulations for the Ca-doped NdBaInO_4_ specified two major vacancy migration ways, respectively, via one intraslab way along the *b*-axis and one interslab way along the *c*-axis. As a result, the authors concluded [72] that the Ca^2+^ is optimal dopant for NdBaInO_4_ among Ca^2+^, Sr^2+^, and Ba^2+^-ions.

**Figure 10:**
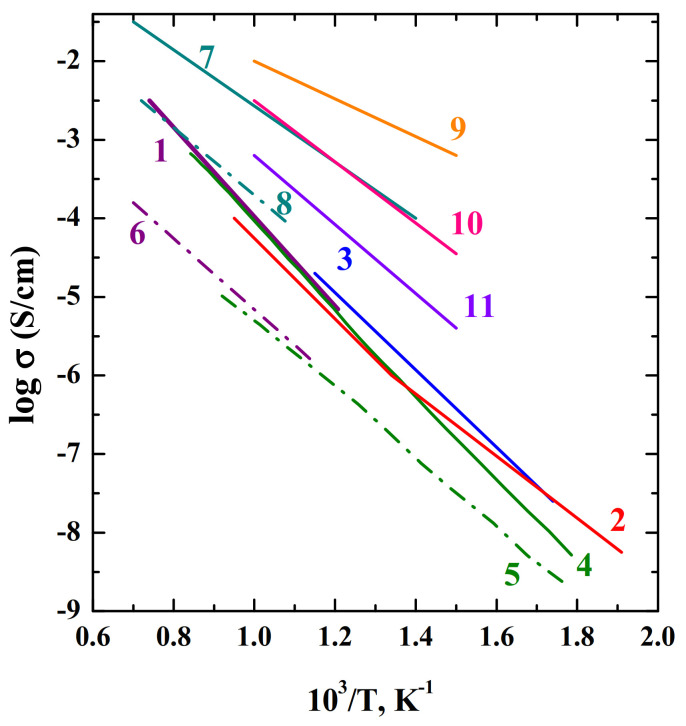
The temperature dependencies of total (solid line) and oxygen-ionic (dash dot line) conductivities of the compositions BaNdInO_4_ (1 and 6) [68], BaNdInO_4_ (2) [82], BaNdInO_4_ (3) [72], BaLaInO_4_ (4 and 5) [76], BaNd_0.9_Sr_0.1_InO_3.95_ (7 and 8) [68], BaNdIn_0.9_Cr_0.1_O_4_ (9) [71], BaNdIn_0.9_Mg_0.1_O_4_ (10) [71], BaNdIn_0.9_Ti_0.1_O_4_ (11) [71].

So, it was found that acceptor doping makes it possible to increase the oxygen-ion conductivity, while in air the samples remain mixed conductors. The ratio of the total and oxygen-ion conductivities is shown in Figure 10. As it can be seen, there is a scatter of data for NdBaInO_4_ in different publications, but, in general, there is a correlation.

#### 4.4.2. Effect of Substitutions on the B-Sites

Effects of substitution at In^3+^-sites in NdBaInO_4_ on O^2−^-conductivity were investigated in NdBaIn_0.9_M_0.1_O_4_ (M = Ce, Ga, Cr, Si, Mg, Zr, Nb, Ta, Ti, and Sn) [71]. As it was shown in Figure 10, the total conductivity in air decreased by doping in the following order: Cr > Mg > Ti > Ce > Nb = Ta = Sn > Zr > Ga >Si. In case of Cr-doping, although highest total conductivity was observed, but, significant electron conductivity appeared and moreover the Cr-containing phase was unstable at low *p*O_2_. Although the Mg-containing sample had a high total conductivity, the ionic conductivity was lower than the Ti and Ce-containing phases. The authors concluded that, in general, doping with higher valence cation is suitable for achieving the higher conductivity. It was found that for increasing oxide ion conductivity the substitution of In^3+^ with Ti^4+^ in NdBaInO_4_ was more effective. As for other phases with the RP-type structure, the high oxide ion conductivity in NdBaInO_4_ could be assigned to the fast oxygen diffusion in rock-salt layer. 

### 4.5. BaLaInO_4_-Based Materials

#### 4.5.1. Effect of Substitutions on the A-Sites

The new La_2_O_3_-containing phases with the composition BaLaInO_4_ were investigated as oxygen-ion and proton conductors since 2018 by our research group. The structure of this phase was described earlier by Titov Y.O. at al. [86].

**Figure 11:**
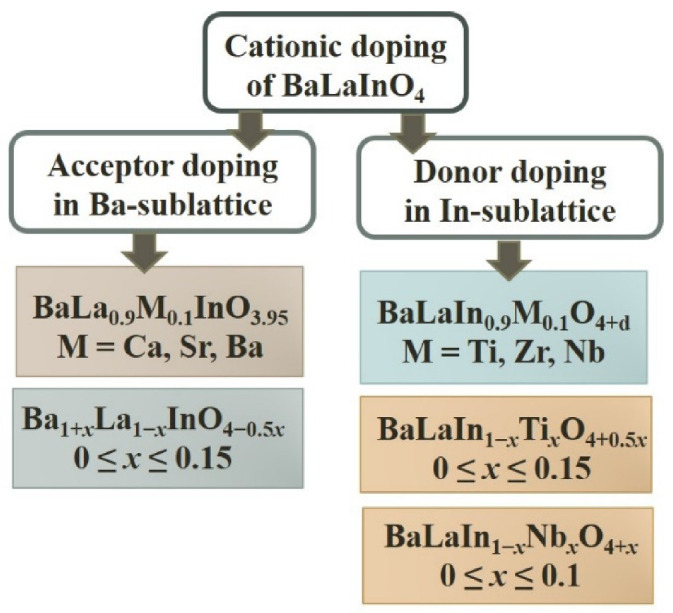
Scheme of acceptor and donor doping of cationic sublattices of BaLaInO_4_.

As it was mentioned above, for undoped phases of the composition BaRInO_4_, the electrical conductivity increased with increasing radius of R^3+^. Following this logic, the phases where R^3+^ = La^3+^ should have the highest oxygen-ionic conductivity. As it is shown in Figure 10, the undoped phase BaLaInO_4_ is characterized by a greater magnitude of the oxygen-ion conductivity in comparison with phase BaNdInO_4_ in the area of lower temperatures (T < 600 °C), and the phase BaNdInO_4_ exhibited higher conductivity at high temperatures. This is explained by the higher activation energies of O^2−^-transport for BaNdInO_4_ (E_a_ = 0.95 [72]) in comparison with BaLaInO_4_ (E_a_ = 0.87 eV [80]). This reason presupposes the promising development of the materials science search for new phases based on BaLaInO_4_ (Figure 11).

**Figure 12:**
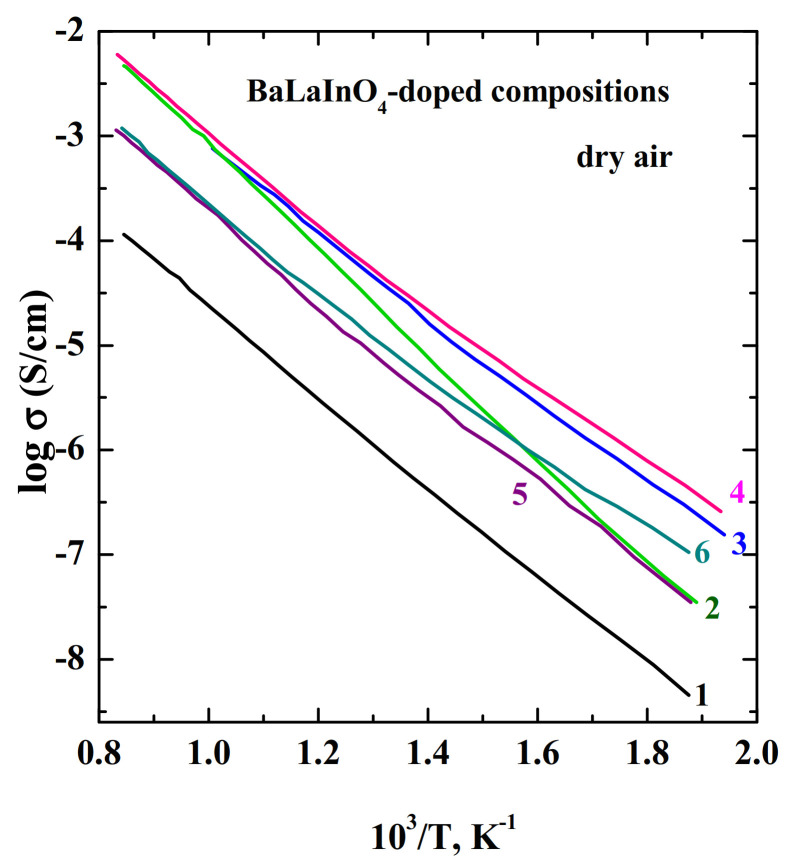
The temperature dependencies of total conductivities in dry air of the compositions BaLaInO_4_ (1) [74], BaLa_0.9_Ca_0.1_InO_3.95_ (2) [74], BaLa_0.9_Sr_0.1_InO_3.95_ (3) [74], Ba_1.1_La_0.9_InO_3.95_ (4) [74], BaLaIn_0.9_Ti_0.1_O_4.05_ (5) [81], BaLaIn_0.9_Nb_0.1_O_4.10_ (6) [84].

The substitution of Ca^2+^, Sr^2+^, Ba^2+^- ions for La^+3^ ion led to the increase in the cell volumes and Ba^2+^-doped sample showed more significant increase in parameters and cell volume [76]. The ionic conductivities of the doped phases increase in the order of BaLa_0.9_Ca_0.1_InO_3.95_−BaLa_0.9_Sr_0.1_InO_3.95_−BaLa_0.9_Ba_0.1_InO_3.95_, i.e., in the order of increasing the ionic radius of dopants (Figure 12). The Nd-phase BaNdInO_4_ doped with M^2+^-ions showed another trend in comparison with the BaLaInO_4_, as it was discussed above. The authors [76] discuss other reasons for the increase in oxide-ion conductivity. The increase in the lattice volume and the lattice parameters reduces the metal−O^2−^ bonding, and, as a consequence, increases the oxygen mobility. In doped BaLaInO_4_ the activation energy of oxide-ion conductivity decreased in the order of Ca^2+^ (0.86 eV)—Sr^2+^ (0.85 eV)—Ba^2+^ (0.82 eV). So, Ba^2+^ is the most suitable dopant on the La^3+^ sites.

**Figure 13:**
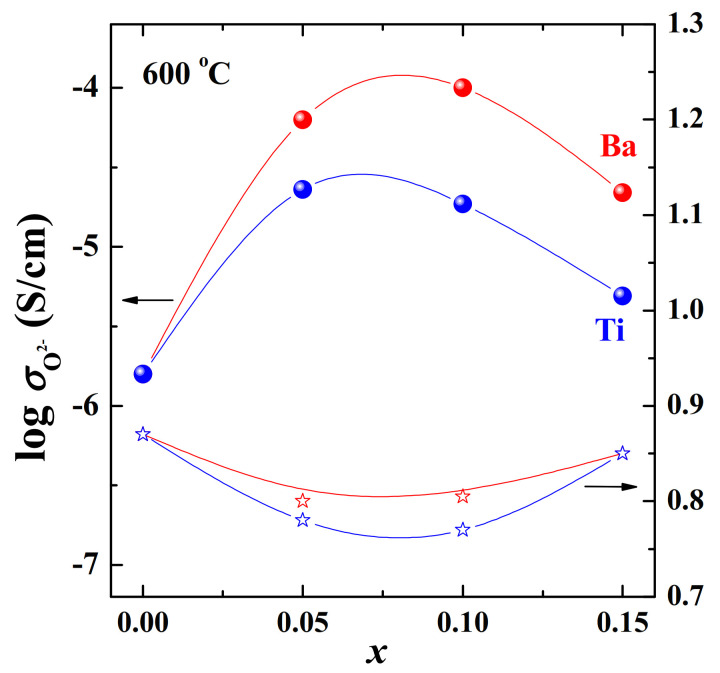
The concentration dependencies of oxygen-ionic conductivities (filled symbols) and activation energies (open symbols) for the solid solutions Ba_1+*x*_La_1−*x*_InO_4−0.5*x*_ (red) [79] and BaLaIn_1−*x*_Ti*_x_*O_4+0.5*x*_ (blue) [80] at 600 °C.

**Figure 14:**
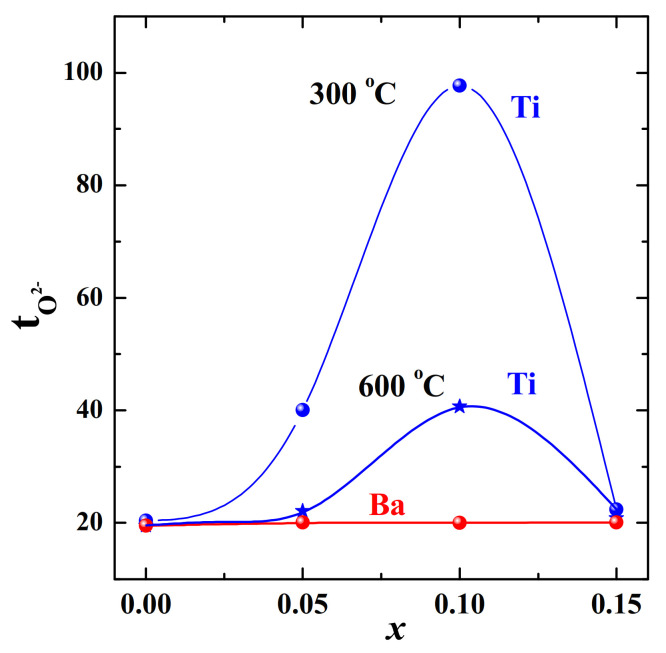
The concentration dependencies of oxygen-ionic transport numbers for the solid solution Ba_1+*x*_La_1−*x*_InO_4−0.5*x*_ (red) [79] at 600 °C and for the solid solution BaLaIn_1−*x*_Ti*_x_*O_4+0.5*x*_ (blue) [80] at 300 and 600 °C.

The solid solution Ba_1+*x*_La_1–*x*_InO_4–0.5*x*_ (0 ≤ *x* ≤ 0.15) was investigated in [79] and summarizing these results, it can be concluded that (Figure 13 and Figure 14):

− Increasing oxygen vacancies due to the M^2+−^additions (Ba^2+^, as an example) results in enhancement of oxygen-ion conductivity;
− Oxygen migration in RP-phases is strongly dependent on the dopant concentrations, there is a narrow range of compositions for increasing conductivity *x* ≤ 0.10;
− High concentrations of dopant (Ba^2+^ *x* ≥ 0.10) lead to interaction of the defects and decrease in the oxygen ion conductivity.

#### 4.5.2. Effect of Substitutions on the B-Sites

The solid solution BaLaIn_1–*x*_Ti*_x_*O_4+0.5*x*_ (0 ≤ *x* ≤ 0.15) was investigated in [80]. The introduction of ions with a smaller ionic radius ($r_{{\mathrm{Ti}}^{4+}}$ = 0.605 Å, $r_{{\mathrm{In}}^{3+}}$ = 0.80 Å [87]) should lead to a decrease of cell parameters, however, decreasing was observed for *c*-parameter, but *a* and *b*-parameters as well as unit cell volume increased with dopant concentration. The observed increase in *a* and *b*-parameters and cell volume may be a consequence of the incorporation of the oxygen interstitials in the rock-salt layers.

The Ti^4+^-doping of BaLaInO_4_ led to the increase in the O^2−^-conductivities up to ~1–1.5 orders of magnitude. The concentration dependencies of the oxygen-ionic conductivity and the temperature dependences of the ionic transport numbers are shown in Figure 13 and Figure 14. The sample BaLaIn_0.9_Ti_0.1_O_4.05_ (*x* = 0.10) exhibited nearly pure oxygen-ionic conduction at T ≤ 400 °C. Comparison of the activation energies of oxygen-ion transport with acceptor-doped phases showed that Ti^4+^-substituted phases had lower activation energies (~0.77 eV) [87].

The Zr^4+^ and Nb^5+^-doped samples showed similar conductivity values in comparison with the Ti^4+^-doped phase [78,84]. The oxygen-ionic conductivity of Nb^5+^-doped phase BaLaIn_0.9_Nb_0.1_O_4.1_ was higher than for undoped composition BaLaInO_4_ by ~1 order of magnitude and the conductivity values for doped sample were comparable and slightly lower than for Ti^4+^-doped composition (Figure 12). So, the appearance of oxygen interstitials can lead to enhancement of the oxygen-ion conductivity of BaLaInO_4._

**Figure 15:**
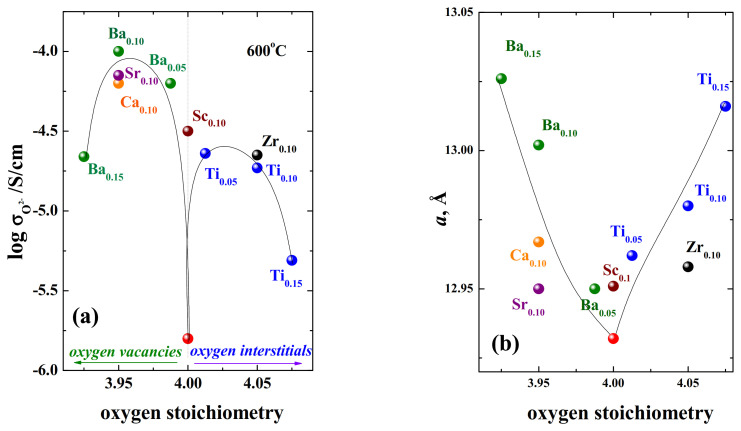
The values of oxygen-ionic conductivity (**a**) and lattice parameter *a* (**b**) vs. oxygen stoichiometry in doped compositions based on BaLaInO_4_: BaLaIn_0.9_Sc_0.1_O_4_ (Sc_0.1_) [83], BaLaInO_4_ (red symbol) [74], Ba_1.05_La_0.95_InO_3.975_ (Ba_0.05_) [79], Ba_1.1_La_0.9_InO_3.95_ (Ba_0.1_) [79], Ba_1.15_La_0.85_InO_3.925_ (Ba_0.15_) [79], BaLa_0.9_Ca_0.1_InO_3.95_ (Ca_0.1_) [74], BaLa_0.9_Sr_0.1_InO_3.95_ (Sr_0.1_) [74], BaLaIn_0.95_Ti_0.05_O_4.025_ (Ti_0.05_) [80], BaLaIn_0.9_Ti_0.1_O_4.05_ (Ti_0.1_) [80], BaLaIn_0.85_Ti_0.15_O_4.075_ (Ti_0.15_) [80], BaLaIn_0.9_Zr_0.1_O_4.05_ (Zr_0.1_) [78].

It is of interest to compare the phases with higher conductivity with an oxygen deficiency and with an oxygen excess in order to understand—Is the oxygen mobility in deficient phases faster than in excess phases? Comparison of oxygen-ion conductivities is shown in Figure 15a. The dependence of the lattice parameters of *a* vs. oxygen nonstoichiometry is also shown (Figure 15b). As can be seen, the *a*-lattice parameters changed significantly upon doping. Comparing the ionic conductivities of the acceptor-doped phases (Ca^2+^, Sr^2+^, Ba^2+^), it should be said that their conductivities were higher than for Ti^4+^, Zr^4+^, Nb^5+^-doped samples. Although, it should be emphasized that both methods of doping can significantly increase the oxygen-ion conductivity. It should also be noted that the phases with the largest lattice expansion have the highest values of the oxygen-ionic conductivity. In this regard, phases with isovalent doping (that is, nominally stoichiometric RP oxides) may be of interest in order to find out, which factor most significantly affects the oxygen mobility in RP-phases—defectiveness or geometric parameters? Such studies have not been carried out in practice, but there is work on the Sc^3+^-doped phase BaLaInO_4_ [83]. The data on the oxygen-ionic conductivity of this phase are also shown in Figure 15. As it can be seen, isovalent doping can also significantly affect oxygen-ion transport. That is the enhancement of the oxygen-ion conductivity can be due to a change in the geometrical factor, and not only in the concentration of the defects. 

Thus, both the factors, defect concentration and geometric factor, have an apparent influence on oxygen ion migration. Which factor is the main one is not yet clear. Since such layered systems with one salt block and one perovskite block are very labile and adapt a large variations of cation substitutions, it is possible that a combination of the optimal size of the salt block and the required concentration of defects will be the most favorable factor for increasing the oxygen-ion conductivity.

### 4.6. BaGdInO_4_-Based Materials

**Figure 16:**
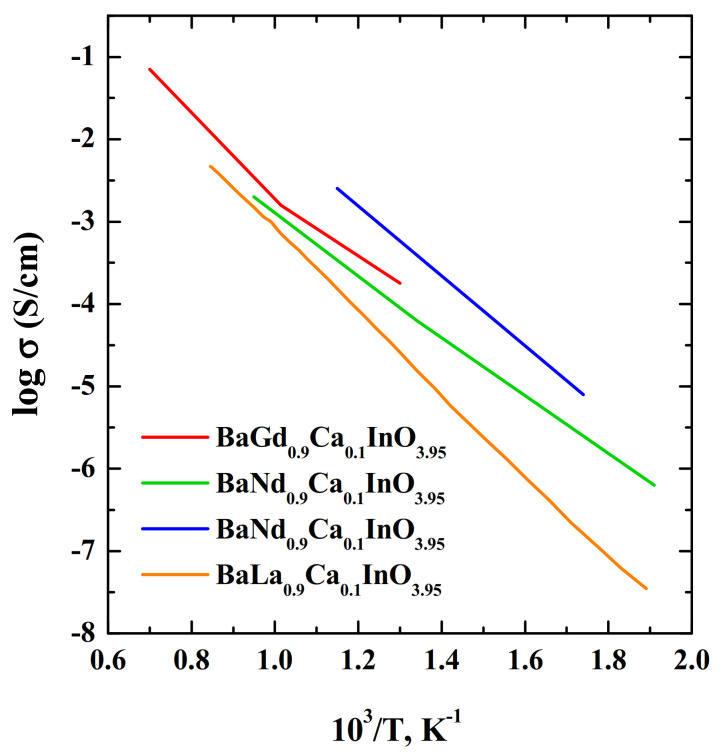
The temperature dependencies of electrical conductivities of the compositions BaGd_0.9_Ca_0.1_InO_3.95_ (red) [115], BaNd_0.9_Ca_0.1_InO_3.95_ (green) [82], BaNd_0.9_Ca_0.1_InO_3.95_ (blue) [72], BaLa_0.9_Ca_0.1_InO_3.95_ (orang) [76].

Recently H.Yaguchi et al. [115] reported the new compounds BaGdInO_4_ and BaGd_0.9_A_0.1_InO_3.95_ (A = Mg, Ca, Sr). These phases belong to a new structure family of oxide-ion conductors and are quite different from those of the monoclinic BaRInO_4_-based compounds. New phases have an orthorhombic *Pnma* Ba_2_Y_2_CuPtO_8_-type structure consisting of square pyramid InO_5_, octahedron InO_6_, (Gd,A)O_7_ polyhedron (monocapped trigonal prism) and Ba cation. The oxide-ion conductivity of BaGd_0.9_Ca_0.1_InO_3.95_ was app. 400 times higher than that of BaGdInO_4_ at 400 °C, as a result of oxygen vacancy formation [115]. The oxide-ion conductivity of BaGd_0.9_Ca_0.1_InO_3.95_ was comparable to those of BaNdInO_4_ -based materials (Figure 16). The bulk conductivity of BaGd_0.9_Ca_0.1_InO_3.95_ was 1.3 × 10^−3^ S/cm at 700 °C and *p*O_2_ = 10^−4^ atm. The authors suggested that the conductivities of BaGdInO_4_-based materials can be improved by various doping. This conclusion is quite justified, since a comparison of the Ca^2+^-doped compositions for the Gd, Nd, La-containing phases confirms this statement (Figure 16).

#### Concluding Remarks

Thus, undoped BaRInO_4_ phases are insulator in nature. The ability of these phases to adapt a wide range of oxygen stoichiometry due to both the deficiency and the excess of oxygen during acceptor or donor doping, respectively, makes it possible to significantly increase the oxygen-ion conductivity. The comparison of the electrical conductivity values is presented in the Table 3. However, in air, the doped phases also retain some level of electronic conductivity. In the recent years researchers have mainly focused attention on doped BaNdInO_4_ and BaLaInO_4_, that is on phases with the largest size of the R^3+^ ion. The effect of cation substitutions on oxygen migration is less understood, compared with the perovskite ABO_3_, since the number of the systems studied is still quite limited. The doped BaRInO_4_ phases are promising and for further improvement these materials need more investigations.

The approach, promising for further investigations, is finding the optimal size of the rock-salt block for oxygen migration with the changing nature of the A- and B-site cations.

## 5. Protonic Conductivity in the Layered Perovskite-Related Materials

For ordinary perovskites, the dissociative incorporation of water (in other words hydration) leads to the formation of protonic defects. This process takes place due to the presence of oxygen vacancies, which may be obtained by acceptor-doping of the oxide. The oxygen vacancies can be replaced by protons (OH^−^) when treated in water vapor at some temperatures, and the proton is attracted to the electron cloud of an oxide ion, and forms hydroxide ion defects (Equation (5)). It is currently an established fact that the migration of protons in perovskites occurs according to the Grotthuss mechanism [116]. The proton rotates around the oxygen and diffuses by jumping to a neighboring oxygen atom.

As shown in Section 3, for layered perovskites the introduction of water is not due to the presence of oxygen vacancies as for ordinary perovskites. Not only the defect structure of the oxide, but mainly the size of the rock-salt layers determines the degree of hydration, which happens according to Equation (7). Therefore, the mechanisms of proton migration will differ from ordinary perovskites. In this respect neutron diffraction techniques will be helpful to provide detailed information about localization of protons and further understanding the mechanism of the proton diffusion. However, these studies are quite rare and the understanding of the mechanism of proton migration has not yet been conclusively established in RP-perovskites.

First investigation of proton transport in the layered perovskites was performed for the compositions Pr_1−*x*_M_1+*x*_InO_4_ (M = Ba^2+^, Sr^2+^; *x* = 0, 0.1) [117]. It was proved that these materials had proton conductivity but oxide-ionic conductivity was extremely low. In contrast, the Pr_1−*x*_Ba_1+*x*_InO_4_ materials exhibited both proton and oxide-ionic conductivity, and with increasing temperature, the proton transport number decreased, and oxide-ionic transport number increased. Both materials seem suitable for operation of proton-conducting solid oxide fuel cells (PC-SOFCs) at targeted temperature from 500 to 700 °C.

**Figure 17:**
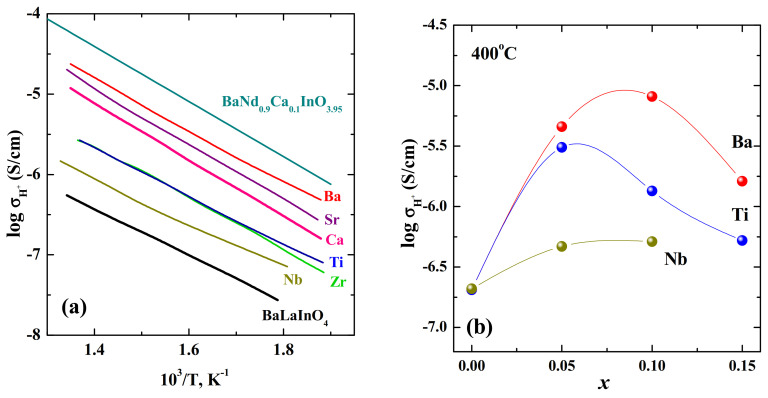
The temperatures dependencies of protonic conductivities for the compositions BaLaInO_4_ [76], BaLa_0.9_Ca_0.1_InO_3.95_ (Ca) [74], BaLa_0.9_Sr_0.1_InO_3.95_ (Sr) [74], Ba_1.1_La_0.9_InO_3.95_ (Ba) [74], BaLaIn_0.9_Ti_0.1_O_4.05_ (Ti) [78], BaLaIn_0.9_Zr_0.1_O_4.05_ (Zr) [78], BaLaIn_0.9_Nb_0.1_O_4.10_ (Nb) [84], BaNd_0.9_Ca_0.1_InO_3.95_ [82] (**a**); and the concentration dependencies of protonic conductivities for the solid solutions Ba_1+*x*_La_1−*x*_InO_4−0.5*x*_ (red) [79], BaLaIn_1−*x*_Ti*_x_*O_4+0.5*x*_ (blue) [80] and BaLaIn_1−*x*_Nb*_x_*O_4+*x*_ (dark yellow) (**b**) [84].

The most complete investigations for the compositions based on BaLaInO_4_ layered perovskite were made. The first article concerning protonic conductivity in the doped compositions based on BaLaInO_4_ was published in the 2018 year [85], and the over ten works appeared till now [76,78,79,80,81,83,84,85,88,89,101]. Figure 17a represents the temperature dependencies of proton conductivities for acceptor BaLa_0.9_M_0.1_InO_3.95_ (M = Ca, Sr, Ba) and donor-doped BaLaIn_0.9_M_0.1_O_4+d_ M = Ti^4+^, Zr^4+^, Nb^5+^ compositions. As can be seen, the doping leads to the increase in the protonic conductivity values up to 1.5 orders of magnitude. However, some regularities can be determined. First, the values of protonic conductivity of acceptor-doped compositions were higher than the conductivity of donor-doped samples with the same dopant concentration. As it was shown earlier, the oxygen-ionic conductivity for these compositions were characterized by the same tendency. Obviously, for the layered perovskites as well as for ordinary perovskites, the dynamics of the oxygen sublattice affects the mobility of protons, and the relationships of protonic transport correlate with the relationships of oxygen ion transport. Second, the increase in the protonic conductivity among compositions with the same type of oxygen defects (oxygen vacancy or oxygen interstitial) correlates with an increase in the amount of water uptake during hydration, i.e., with proton concentration. Therefore, both factors including concentration of protonic species and their mobility affect significantly to the values of protonic conductivity.

To understand the role of change in the concentration of point defects, the conductivity values for the compositions with different concentration of the same dopant must be considered. Figure 17b represents the concentration dependencies of protonic conductivities for the solid solutions Ba_1+*x*_La_1−*x*_InO_4−0.5*x*_, BaLaIn_1−*x*_Ti*_x_*O_4+0.5*x*_, and BaLaIn_1−*x*_Nb*_x_*O_4+*x*_ at 400 °C. For all solid solutions, increasing the dopant concentration leads to the increase in the unit cell volume and, consequently, to the increase of the proton concentration. Then, the increase in the protonic conductivity can be expected. However, the obtained results do not match this assumption. As can be seen (Figure 17b), the maximum of protonic conductivity is observed for small (0.05−0.10) dopant concentrations. The subsequent increase in dopant concentration leads to the decrease in the conductivity values due to the formation of proton-aggregating clusters:

(9) $$M_{A}^{\prime }+{(\mathrm{OH})}_{o}^{•}\to {\left(M_{A}^{\prime }\cdot {(\mathrm{OH})}_{o}^{•}\right)}^{\times }$$

(10) $$M_{B}^{•}+{(\mathrm{OH})}_{i}^{\prime }\to {\left(M_{B}^{•}\cdot {(\mathrm{OH})}_{i}^{\prime }\right)}^{\times }$$

In other words, the increase in the acceptor/donor dopant concentration leads to the trapping of protons for both acceptor- and donor-doped solid solutions despite the different types of oxygen defects.

**Figure 18:**
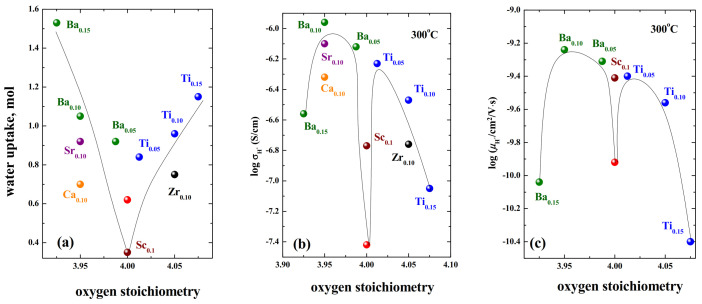
The water uptake (**a**), protonic conductivity (**b**) and protonic mobility (**c**) vs. oxygen stoichiometry in doped compositions based on BaLaInO_4_: BaLaIn_0.9_Sc_0.1_O_4_ (Sc_0.1_) [83], BaLaInO_4_ (red symbol) [74], Ba_1.05_La_0.95_InO_3.975_ (Ba_0.05_) [79], Ba_1.1_La_0.9_InO_3.95_ (Ba_0.1_) [79], Ba_1.15_La_0.85_InO_3.925_ (Ba_0.15_) [79], BaLa_0.9_Ca_0.1_InO_3.95_ (Ca_0.1_) [74], BaLa_0.9_Sr_0.1_InO_3.95_ (Sr_0.1_) [74], BaLaIn_0.95_Ti_0.05_O_4.025_ (Ti_0.05_) [80], BaLaIn_0.9_Ti_0.1_O_4.05_ (Ti_0.1_) [80], BaLaIn_0.85_Ti_0.15_O_4.075_ (Ti_0.15_) [80], BaLaIn_0.9_Zr_0.1_O_4.05_ (Zr_0.1_) [78].

In this way, for the doped compositions based on BaLaInO_4_ two factors (concentration and mobility of protons) play a significant role in the protonic transport. In the area of “low” dopant concentration, the proton conductivity increases due to increase in both concentration of current carriers and their mobility (Figure 18). In the area of “big” dopant concentration, the decrease in the proton mobility plays more significant role than the increase in the proton concentration. Consequently, obtaining high-conductive protonic electrolytes with layered perovskite structure is needed for the optimal combination of both factors. It should be noted that all acceptor- and donor-doped samples based on BaLaInO_4_ samples are ~ 90−98% proton conductors under wet air below 400 °C, which is good characteristic with respect to their possible application as an electrolytic materials in H-SOFCs.

In the last three years, several articles concerning protonic conductivity in other layered perovskites were published. The donor doping in the A-sublattice of A^II^LnInO_4_ was investigated for the layered perovskite SrLaInO_4_ [77]. The presence of the protons in the structure of Ba_x_Sr_0.8−*x*_La_1.2_InO_4+d_ was shown by the neutron powder diffraction method. However, the conductivity measurements were performed without controlling the water partial pressure, and the protonic conductivity values were not obtained. The compositions with acceptor doping in the A-sublattice for layered perovskites BaNdInO_4_ [82] and BaNdScO_4_ [118] were obtained. For solid solutions BaNd_1−*x*_Ca*_x_*InO_4−0.5*x*_ and BaNd_1−*x*_Ca*_x_*ScO_4−0.5*x*_ (*x* = 0.1; 0.2 for both solid solutions) it was shown that the doping leads to the increase in the protonic conductivity values in comparison with undoped samples; and the compositions with *x* = 0.2 demonstrated the highest protonic conductivity. However, the systematic investigations of the effect of nature and concentration of dopant on the protonic conductivity were not performed for these layered perovskites.

The comparison of proton conductivity values for Ca^2+^-doped composition based on BaNdInO_4_ [82] with values for doped compositions based on BaLaInO_4_ is presented in the Figure 17a. As can be seen, the composition BaNd_0.9_Ca_0.1_InO_3.95_ had higher conductivity values compared with the values for the composition of more conductive BaLa_0.9_Ba_0.1_InO_3.95_. However, the proton transport numbers for BaNd_0.9_Ca_0.1_InO_3.95_ did not exceed 0.57 at the low temperatures (250−475 °C). At the same time, the composition BaLa_0.9_Ba_0.1_InO_3.95_ was characterized by almost fully protonic transport (95−98% below 400 °C) despite slightly lower proton conductivity values.

Therefore, the layered perovskites AA′BO_4_ are the new and promising class of proton conductors. The nature of cations of their constituent as well as the nature and concentration of dopants strongly affect the values of proton conductivity and the proton transport numbers. Obviously, the further materials research will allow to obtain the novel compositions with layered perovskite structure, characterized by high proton conductivity and fully protonic transport at the same time.

## 6. Conclusions and Outlook

**Figure 19:**
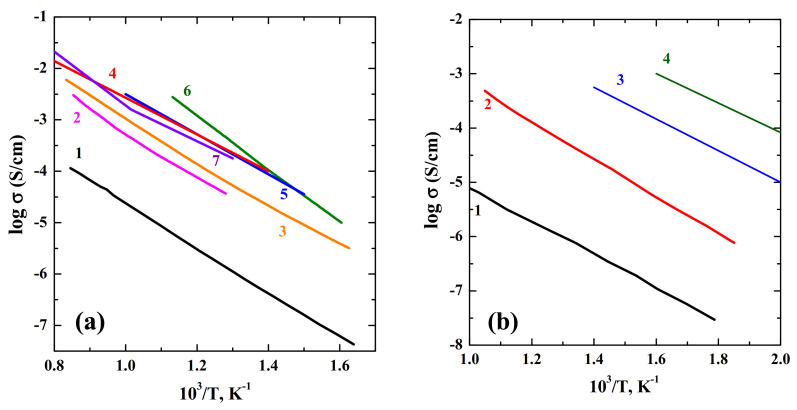
The temperature dependencies of conductivity under dry air (**a**): BaLaInO_4_ (1) [76], Sr_0.6_Ba_0.2_La_1.2_InO_4+δ_ (2) [74], Ba_1.1_La_0.9_InO_3.95_ (3) [76], BaNd_0.9_Sr_0.1_InO_3.95_ (4) [68], BaNdIn_0.9_Mg_0.1_O_4_ (5) [71], ZrO_2_ (8 mol% Y_2_O_3_) [74] (6), BaGd_0.9_Ca_0.1_InO_3.95_ [115] (7) and under wet air (**b**): BaLaInO_4_ (1) [76], Ba_1.1_La_0.9_InO_3.95_ (2) [76], SrCeO_3_(10 mol% Y_2_O_3_) (3) [119], BaCeO_3_ (10 mol% Y_2_O_3_) (4) [119].

The materials research over the past few years has highlighted one more application area of layered perovskites. Besides superconductors, magnetoresistors, dielectrics, thermoelectrics, phosphors, and photocatalysts, the compositions with layered PR-structure can be used as the oxide-ions and proton conductors. The most investigated materials with general formula A^II^LnInO_4_ are the compositions BaNdInO_4_ and BaLaInO_4_, which demonstrate mixed hole-ionic nature of conductivity in dry air. Acceptor and donor doping leads to a significant increase (up to ~1.5 orders of magnitude) of conductivity. However, in dry air, the undoped and doped phases also retain some level of hole conductivity. Despite this, the compositions with sufficiently high conductivity values were obtained (Figure 19a). The interaction of these materials with water vapor leads to the dissociative dissolution of water molecules and the localization of hydroxyl groups within the rock-salt layers. The amount of water uptake increases with the increase of unit cell volume and it is not determined by the concentration of oxygen defects in the structure. The water uptake for doped compositions based on BaLaInO_4_ is up to 1.5 mol H_2_O per formula unit, which is much bigger than for known perovskite-related materials. However, the proton concentration in the structure is not a main factor determining the high proton conductivity. For the layered perovskites based on BaLaInO_4_ it was shown that the presence of “big” dopant content (>0.1 mol) led to the decrease in the proton mobility due to the appearance of clusters with lower mobility. Consequently, the task of creation of novel highly conductive protonic electrolytes requires the complex approach including choosing the nature and the ratio of cations in the structure (Figure 19b).

In the past years, the need of developing new materials suitable for using in various electrochemical devices keeps growing. From electrocatalysts [120] and MIEC membranes [121] to SOFCs [122], PCFCs, and PCECs [123], every branch of energy application sciences requires novel and highly effective materials for creation of advanced devices and technologies. The active growth of investigation of cathode materials with layered perovskite structure [46,47,48,49,50] makes the task of creating electrochemical sources with the same type of structure of electrolyte more relevant. Sure enough, proton-conducting layered perovskites must take a significant place in the roadmap of future inorganic materials science.

## Figures and Tables

**Table 1:** Some compositions with layered perovskite structure, obtained by Titiov et al. [86], the cation size ratio is indicated in brackets.

Samples with Main Phase with K_2_NiF_4_ Structure and Some Unidentifiable Impurity	Single-Phase Samples with Orthorhombic Structure (s.g. *Pbca*)	Samples with Main Phase with Rhombic Perovskite Structure and Some Unidentifiable Impurity
SrNdInO_4_ (1.545) SrSmInO_4_ (1.526)	BaLaInO_4_ (1.678) SrLaInO_4_ (1.578) SrPrInO_4_ (1.555)	CaLaInO_4_ (1.497) BaPrInO_4_ (1.655) BaNdInO_4_(1.645)

**Table 2:** Some structural characteristics of layered perovskites, obtained by Titiov et al. [86].

Composition	R¯ARB	a, Å	Δ (A_(1),_A_(2)_)O_9_ × 10^−4^	Bond Length A_(1),_A_(2)_−O2 (Interlayer Space), Å	$r_{A\left(1\right)}$, Å	$r_{A\left(2\right)}$, Å
BaLaInO_4_	1.678	12.933(3)	154	2.341	1.47	1.216
SrLaInO_4_	1.578	12.594(2)	192	2.382	1.31	1.216
SrPrInO_4_	1.555	12.474(4)	249	2.323	1.31	1.179

**Table 3:** The comparison of the electrical conductivity values of phases with Ruddlesden–Popper structure.

Composition	Values of Electrical Conductivity under Dry Air at 500 °C, S/cm	Ref.
SrLaInO_4_	4.1 × 10^−6^	[70]
SrLaInO_4_	1.0 × 10^−4^	[66]
SrLaIn_0.8_Zr_0.2_O_4+d_	2.0 × 10^−4^	[70]
Sr_1.1_La_0.9_InO_3.95_	1.5 × 10^−5^	[66]
Sr_1.2_La_0.8_InO_3.90_	4.5 × 10^−6^	[66]
BaNdInO_4_	1.4 × 10^−7^	[73]
BaNdInO_4_	1.0 × 10^−7^	[72]
BaNdInO_4_	6.2 × 10^−5^	[82]
BaNdIn_0.9_Cr_0.1_O_4_	2.2 × 10^−4^	[71]
BaNd_0.9_Sr_0.1_InO_3.95_	3.3 × 10^−5^	[68]
BaNd_0.9_Ca_0.1_InO_3.95_	5.0 × 10^−4^	[72]
BaNdIn_0.9_Mg_0.1_O_4_	3.5 × 10^−6^	[71]
BaNdIn_0.9_Ti_0.1_O_4_	1.6 × 10^−6^	[71]
BaGdInO_4_	6.3 × 10^−8^	[72]
BaGd_0.9_Ca_0.1_InO_3.95_	2.0 × 10^−4^	[115]
BaLaInO_4_	5.0 × 10^−5^	[76]
Ba_1.1_La_0.9_InO_3.95_	2.5 × 10^−5^	[74]
BaLa_0.9_Sr_0.1_InO_3.95_	1.3 × 10^−5^	[74]
BaLa_0.9_Ca_0.1_InO_3.95_	7.9 × 10^−6^	[74]
BaLaIn_0.9_Nb_0.1_O_4.10_	1.7 × 10^−4^	[84]
BaLaIn_0.9_Ti_0.1_O_4.05_	1.0 × 10^−4^	[81]
BaYInO_4_	1.7 × 10^−3^	[72]
BaErInO_4_	2.2 × 10^−4^	[72]

## References

[1] Watts N., Amann M., Arnell N., Montgomery H., Costello A. (2021). The 2020 report of The Lancet Countdown on health and climate change: Responding to converging crises. Lancet.

[2] Corvalan C., Prats E.V., Sena A., Varangu L., Vinci S. (2020). Towards Climate Resilient and Environmentally Sustainable Health Care Facilities. Int. J. Environ. Res. Public Health.

[3] Nejat Veziroglu T. (2012). Conversion to hydrogen economy. Energy Procedia.

[4] Duan C., Huang J., Sullivan N., O’Hayre R. (2020). Proton-conducting oxides for energy conversion and storage. Appl. Phys. Rev..

[5] Colomban P. (2019). Proton conductors and their applications: A tentative historical overview of the early researches. Solid State Ion..

[6] Medvedev D. (2019). Trends in research and development of protonic ceramic electrolysis cells. Int. J. Hydrogen Energy.

[7] Shim J.H. (2018). Ceramics breakthrough. Nat. Energy.

[8] Meng Y., Gao J., Zhao Z., Amoroso J., Tong J., Brinkman K.S. (2019). Review: Recent progress in low-temperature proton-conducting ceramics. J. Mater. Sci..

[9] Kim J., Sengodan S., Kim S., Kwon O., Bud Y., Kim G. (2019). Proton conducting oxides: A review of materials and applications for renewable energy conversion and storage. Renew. Sustain. Energy Rev..

[10] Zvonareva I., Fu X.-Z., Medvedev D., Shao Z. (2021). Electrochemistry and energy conversion features of protonic ceramic cells with mixed ionic-electronic electrolytes. Energy Environ. Sci..

[11] Medvedev D.A. (2021). Current drawbacks of proton-conducting ceramic materials: How to overcome them for real electrochemical purposes. Curr. Opin. Green Sustain. Chem..

[12] von Balz D., Plieth K. (1955). Die Struktur des Kaliumnickelfluorids, K_2_NiF_4_. Z. Für Elektrochem..

[13] Ruddlesden S.N., Popper P. (1957). New compounds of the K_2_NiF_4_ type. Acta Cryst..

[14] Momma K., Izumi F. (2011). VESTA 3 for three-dimensional visualization of crystal, volumetric and morphology data. J. Appl. Crystallogr..

[15] Ruddlesden S.N., Popper P. (1958). The compound Sr_3_Ti_2_O_7_ and its structure. Acta Cryst..

[16] Wells A.F. (1984). Structural Inorganic Chemistry.

[17] Ganguly P., Rao C.N.R. (1984). Crystal Chemistry and Magnetic Properties of Layered Metal Oxides Possessing the K2NiF4 or Related Structures. J. Solid State Chem..

[18] Ganculi D. (1979). Cationic radius ratio and formation of K_2_NiF_4_-type compounds. J. Solid State Chem..

[19] Bednorz J.G., Müller K.A. (1986). Possible high Tc superconductivity in the Ba-La-Cu-O system. Z. Phys. B—Condens. Matter.

[20] Hirayama T., Nakagawa M., Sumiyama A., Oda Y. (1998). Superconducting properties in La_2_CuO_4+δ_ with excess oxygen. Phys. Rev. B.

[21] Tholence J.L. (1987). Superconductivity of La_2_CuO_4_ and YBa_2_Cu_3_O_7_. Phys. B+C.

[22] Suter A., Logvenov G., Boris A.V., Baiutti F., Wrobel F., Howald L., Stilp E., Salman Z., Prokscha T., Keimer B. (2018). Super-conductivity drives magnetism in δ-doped La_2_CuO_4_. Phys. Rev. B.

[23] Bates F.E., Eldridge J.E. (1989). Normal modes of tetragonal La_2_NiO_4_ and La_2_CuO_4_, isomorphs of the hight Tc superconductor La_2_-_x_Sr_x_CuO_4_. Solid State Commun..

[24] Burns G., Dacol F.H., Kliche G., Konig W., Shafer M.W. (1988). Raman and infrared studies of Sr_2_TiO_4_: A material isomorphic to (La,Sr)2CuO4 superconductors. Phys. Rev. B.

[25] Putilin S.N., Antipov E.V., Chmaissem O., Marezio M. (1993). Superconductivity at 94 K in HgBa_2_CuO_4+δ_. Nature.

[26] Qiu D., Gong C., Wang S., Zhang M., Yang C., Wang X., Xiong J. (2021). Recent Advances in 2D Superconductors. Adv. Mater..

[27] Jin S., Tiefel T.H., McCormack M., Fastnacht R.A., Ramesh R., Chen L.H. (1994). Thousandfold change in resistivity in magnetore-sistive La-Ca-Mn-O films. Science.

[28] Moritomo Y., Asamitsu A., Kuwahara H., Tokura Y. (1996). Giant magnetoresistance of manganese oxides with a layered perovskite structure. Nature.

[29] Salamon M.B., Jaime M. (2001). The physics of manganites: Structure and transport. Rev. Modern. Phys..

[30] Mootabian M., Ghorbani S.R., Kompany A., Abrishami M.E. (2021). Effect of Fe and Co doping on structural and electrical properties of La_0.5_Sr_1.5_MnO_4_ layered-structure and the corresponding La_0.5_Sr_0.5_MnO_3_ perovskite. J. Alloys Compd..

[31] Pajaczkowska A., Gloubokov A. (1998). Synthesis, growth and characterization of tetragonal ABCO4 crystals. Prog. Cryst. Growth Charact. Mater..

[32] Liu X.Q., Chen X.M., Xiao Y. (2003). Preparation and characterization of LaSrAlO4 microwave dielectric ceramics. Mater. Sci. Eng. B Solid-State Mater. Adv..

[33] Mao M.M., Chen X.M., Liu X.Q. (2011). Structure and microwave dielectric properties of solid solution in SrLaAlO_4_-Sr_2_TiO_4_ system. J. Am. Ceram. Soc..

[34] Liu B., Li L., Liu X.Q., Chen X.M. (2016). Structural evolution of SrLaAl1-x(Zn0.5Ti0.5)xO4 ceramics and effects on their microwave dielectric properties. J. Mater. Chem. C.

[35] Liu X., He L., Yu M., Zuo R. (2021). Temperature-stable and ultralow-loss (1−x)CaSmAlO_4–x_Sr_2_TiO_4_ microwave dielectric solid-solution ceramics. J. Mater. Sci..

[36] Danielson E., Devenney M., Giaquinta D.M., Golden J.H., Haushalter R.C., McFarland E.W., Poojary D.M., Reaves C.M., Weinberg W.H., Wu X.D. (1998). X-ray powder structure of Sr_2_CeO_4_: A new luminescent material discovered by combinatorial chemistry. J. Mol. Struct..

[37] Li J., Li X., Hu S., Li Y., Hao Y. (2013). Photoluminescence mechanisms of color-tunable Sr_2_CeO_4_:Eu3+, Dy3+ phosphors based on experimental and first-principles investigation. Opt. Mater..

[38] Sahu M., Gupta S.K., Jain D., Saxena M.K., Kadam R.M. (2018). Solid state speciation of uranium and its local structure in Sr2CeO4 using photoluminescence spectroscopy. Spectrochim. Acta A Mol. Biomol. Spectrosc..

[39] Viesca-Villanueva E., Oliva J., Chavez D., Lopez-Badillo C.M., Gomez-Solis C., Mtz-Enriquez A.I., Garcia C.R. (2020). Effect of Yb3+ codopant on the upconversion and thermoluminescent emission of Sr_2_CeO_4_:Er3+, Yb3+ phosphors. J. Phys. Chem. Solids.

[40] Kharton V.V., Viskup A.P., Naumovkh E.N., Marques F.M.B. (1999). Oxygen ion transport in La_2_NiO_4_-based ceramics. J. Mater. Chem..

[41] Kharton V.V., Viskup A.P., Kovalevsky A.V., Naumovich E.N., Marques F.M.B. (2001). Ionic transport in oxy-gen-hyperstoichiometric phases with K_2_NiF_4_-type structure. Solid State Ion..

[42] Bassat J.M., Burriel M., Wahyudi O., Castaing R., Ceretti M., Veber P., Weill I., Villesuzanne A., Grenier J.C., Paulus W. (2013). Anisotropic oxygen diffusion properties in Pr2NiO4+δ and Nd2NiO4+δ single crystals. J. Phys. Chem. C.

[43] Lee D., Grimaud A., Crumlin E.J., Mezghani K., Habib M.A., Feng Z.X., Hong W.T., Biegalski M.D., Christen H.M., Shao-Horn Y. (2013). Strain influence on the oxygen electrocatalysis of the (100)-oriented epitaxial La_2_NiO_4+δ_ thin films at elevated tem-peratures. J. Phys. Chem. C.

[44] Boehm E., Bassat J.M., Dordor P., Mauvy F., Grenier J.C., Stevens P. (2005). Oxygen diffusion and transport properties in non-stoichiometric Ln_2−x_NiO_4+δ_ oxides. Solid State Ion..

[45] Ishihara T., Miyoshi S., Furuno T., Sanguanruang O., Matsumoto H. (2006). Mixed conductivity and oxygen permeability of doped Pr_2_NiO_4_-based oxide. Solid State Ion..

[46] Tarutin A.P., Lyagaeva J.G., Farlenkov A.S., Vylkov A.I., Medvedev D.M. (2019). Cu-substituted La_2_NiO_4+δ_ as oxygen electrodes for protonic ceramic electrochemical cells. Ceram. Int..

[47] Tarutin A.P., Lyagaeva Y.G., Vylkov A.I., Gorshkov M.Y., Vdovin G.K., Medvedev D.A. (2021). Performance of Pr_2_(Ni,Cu)O_4+δ_ elec-trodes in protonic ceramic electrochemical cells with unseparated and separated gas spaces. J. Mater. Sci. Technol..

[48] Tarutin A., Lyagaeva J., Farlenkov A., Plaksin S., Vdovin G., Demin A., Medvedev D. (2019). A Reversible Protonic Ceramic Cell with Symmetrically Designed Pr2NiO4+δ-Based Electrodes: Fabrication and Electrochemical Features. Materials.

[49] Tarutin A., Gorshkov Y., Bainov A., Vdovin G., Vylkov A., Lyagaeva J., Medvedev D. (2020). Barium-doped nickelates Nd_2−x_BaxNiO_4+δ_ as promising electrode materials for protonic ceramic electrochemical cells. Ceram. Int..

[50] Tarutin A., Lyagaeva J., Medvedev D., Bi L., Yaremchenko A. (2021). Recent advances in layered Ln_2_NiO_4+δ_ nickelates: Fundamentals and prospects of their applications in protonic ceramic fuel and electrolysis cells. J. Mater. Chem. A.

[51] Rivas J., Rivas-Murias B., Fondado A., Mira J., Señarís-Rodríguez M.A. (2004). Dielectric response of the charge-ordered two-dimensional nickelate La_1.5_Sr_0.5_NiO_4_. Appl. Phys. Lett..

[52] Liu X.Q., Jia B.W., Yang W.Z., Cheng J.P., Chen X.M. (2010). Dielectric relaxation andpolaronic hopping in Al-substituted Sm_1.5_Sr_0.5_NiO_4_ ceramics. J. Phys. D Appl. Phys..

[53] Jia B.W., Liu X.Q., Chen X.M. (2011). Structure, magnetic and dielectric properties in Mn-substituted Sm_1.5_Sr_0.5_NiO_4_ ceramics. J. Appl. Phys..

[54] Liu G., Chen T.T., Wang J., Liu X.Q., Chen X.M. (2013). Effect of excess oxygen oncrystal structures and dielectric responses of Nd_2_NiO_4+δ_ ceramics. J. Alloys Compd..

[55] Jiang D., Xia Z., Huang S., Yang F., Song Y., Deng H., Zhang X., Niu H., Zeng Z., Cheng C. (2020). Dielectric response and magneto-electric interaction of La_1.67_Sr_0.33_NiO_4_ single crystal. J. Magn. Magn. Mater..

[56] Shimada Y., Miyasaka S., Kumai R., Tokura Y. (2006). Semiconducting ferromagnetic states in La1-xSr1+xCoO4. Phys. Rev. B Condens. Matter..

[57] Chichev A.V., Dlouhá M., Vratislav S., Knížek K., Hejtmánek J., Maryško M., Veverka M., Jirák Z., Golosova N.O., Ko-zlenko D.P. (2006). Structural, magnetic, and transport properties of the single-layered perovskites La_2−x_Sr_x_CoO_4_ (x = 1.0–1.4). Phys. Rev. B Condens. Matter..

[58] Mukherjee R., Dan S., Mukherjee S., Ranganathan R. (2021). Structural and magnetic properties of the layered perovskite system Sr_2−x_Pr_x_CoO_4_ (x = 0.7, 0.9, 1.1). J. Phys. Chem. Solids.

[59] Lee K.H., Kim S.W., Ohta H., Koumoto K. (2006). Ruddlesden-Popper phases as thermoelectric oxides: Nb-doped SrO(SrTiO_3_)n (n = 1, 2). J. Appl. Phys..

[60] Wang Y., Wan C., Zhang X., Shen L., Koumoto K., Gupta A., Bao N. (2013). Influence of excess SrO on the thermoelectric properties of heavily doped SrTiO_3_ ceramics. Appl. Phys. Lett..

[61] Putri Y.E., Said S.M., Refinel R., Ohtaki M., Syukri S. (2018). Low Thermal Conductivity of RE-Doped SrO(SrTiO_3_)1 Ruddlesden Popper Phase Bulk Materials Prepared by Molten Salt Method. Electron. Mater. Lett..

[62] Shi X.-L., Wu H., Liu Q., Zhou W., Lu S., Shao Z., Dargusch M., Chen Z.-G. (2020). SrTiO_3_-based thermoelectrics: Progress and challenges. Nano Energy.

[63] Jia Y., Shen S., Wang D., Wang X., Shi J., Zhang F., Han H., Li C. (2013). Composite Sr_2_TiO_4_/SrTiO_3_(La,Cr) heterojunction based photocatalyst for hydrogen production under visible light irradiation. J. Mater. Chem. A.

[64] Zhang H., Ni S., Mi Y., Xu X. (2018). Ruddlesden-Popper compound Sr2TiO4 co-doped with La and Fe for efficient photocatalytic hydrogen production. J. Catal..

[65] Ziati M., Bekkioui N., Ez-Zahraouy H. (2021). Ruddlesden-Popper compound Sr2TiO4 doped with chalcogens for optoelectronic ap-plications: Insights from first-principle calculations. Chem. Phys..

[66] Kato S., Ogasawara M., Sugai M., Nakata S. (2002). Synthesis and oxide ion conductivity of new layered perovskite La_1−x_Sr_1+x_InO_4−d_. Solid State Ion..

[67] Fujii K., Esaki Y., Omoto K., Yashima M., Hoshikawa A., Ishigaki T., Hester J.R. (2014). New Perovskite-Related Structure Family of Oxide-Ion Conducting Materials NdBaInO_4_. Chem. Mater..

[68] Fujii K., Shiraiwa M., Esaki Y., Yashima M., Kim S.J., Lee S. (2015). Improved oxide-ion conductivity of NdBaInO_4_ by Sr doping. J. Mater. Chem. A.

[69] Troncoso L., Alonso J.A., Aguadero A. (2015). Low activation energies for interstitial oxygen conduction in the layered perovskites La_1+x_Sr_1−x_InO_4+d_. J. Mater. Chem. A.

[70] Troncoso L., Alonso J.A., Fernández-Díaz M.T., Aguadero A. (2015). Introduction of interstitial oxygen atoms in the layered perovskite LaSrIn_1−x_B_x_O_4+δ_ system (B = Zr, Ti). Solid State Ion..

[71] Ishihara T., Yan Y., Sakai T., Ida S. (2016). Oxide ion conductivity in doped NdBaInO_4_. Solid State Ion..

[72] Yang X., Liu S., Lu F., Xu J., Kuang X. (2016). Acceptor Doping and Oxygen Vacancy Migration in Layered Perovskite NdBaInO_4_-Based Mixed Conductors. J. Phys. Chem. C.

[73] Fijii K., Yashima M. (2018). Discovery and development of BaNdInO_4_—A brief review. J. Ceram. Soc. Jpn..

[74] Troncoso L., Mariño C., Arce M.D., Alonso J.A. (2019). Dual Oxygen Defects in Layered La_1.2_Sr_0.8−x_BaxInO_4+d_ (x = 0.2, 0.3) Oxide-Ion Conductors: A Neutron Diffraction Study. Materials.

[75] Tarasova N., Animitsa I. (2015). Protonic transport in oxyfluorides Ba_2_InO_3_F and Ba_3_In_2_O_5_F_2_ with Ruddlesden–Popper structure. Solid State Ion..

[76] Tarasova N., Animitsa I., Galisheva A., Korona D. (2019). Incorporation and Conduction of Protons in Ca, Sr, Ba-Doped BaLaInO_4_ with Ruddlesden-Popper Structure. Materials.

[77] Troncoso L., Arce M.D., Fernández-Díaz M.T., Mogni L.V., Alonso J.A. (2019). Water insertion and combined interstitial-vacancy oxygen conduction in the layered perovskites La_1.2_Sr_0.8−x_BaxInO_4+δ_. New J. Chem..

[78] Tarasova N., Animitsa I., Galisheva A., Pryakhina V. (2020). Protonic transport in the new phases BaLaIn_0.9_M_0.1_O_4.05_ (M = Ti, Zr) with Ruddlesden-Popper structure. Solid State Sci..

[79] Tarasova N., Animitsa I., Galisheva A. (2020). Electrical properties of new protonic conductors Ba_1+x_La_1−x_InO^4−0.5x^ with Ruddlesden-Popper structure. J. Solid State Electrochem..

[80] Tarasova N., Galisheva A., Animitsa I. (2020). Improvement of oxygen-ionic and protonic conductivity of BaLaInO_4_ through Ti doping. Ionics.

[81] Tarasova N., Galisheva A., Animitsa I. (2021). Ba^2+^/Ti^4+^-co-doped layered perovskite BaLaInO_4_: The structure and ionic (O^2−^, H^+^) con-ductivity. Int. J. Hydrogen Energy.

[82] Zhou Y., Shiraiwa M., Nagao M., Fujii K., Tanaka I., Yashima M., Baque L., Basbus J.F., Mogni L.V., Skinner S.J. (2021). Protonic Conduction in the BaNdInO_4_ Structure Achieved by Acceptor Doping. Chem. Mater..

[83] Tarasova N.A., Galisheva A.O., Animitsa I.E., Lebedeva E.L. (2021). Oxygen-Ion and Proton Transport in Sc-Doped Layered Perovskite BaLaInO_4_. Russ. J. Electrochem..

[84] Tarasova N.A., Galisheva A.O., Animitsa I.E., Dmitrieva A.A. (2021). The Effect of Donor Doping on the Ionic (O^2−^, H^+^) Transport in Novel Complex Oxides BaLaIn_1−x_Nb_x_O_4+x_ with the Ruddlesden–Popper Structure Russ. J. Electrochem..

[85] Korona D.V., Obrubova A.V., Kozlyuk A.O., Animitsa I.E. (2018). Hydration and Proton Transport in BaCa_x_La_1−x_InO_4−0.5x_ (x = 0.1 and 0.2) Phases with Layered Structure. Russ. J. Phys. Chem. A.

[86] Tyitov Y.O., Byilyavina N.M., Markyiv V.Y., Slobodyanik M.S., Krajevs’ka Y.A. (2009). Synthesis and crystal structure of BaLaInO_4_ and SrLnInO4 (Ln−La, Pr). Dopov. Natsyional’noyi Akad. Nauk Ukrayini.

[87] Shannon R.D. (1976). Revised effective ionic radii and systematic studies of interatomic distances in halides and chalcogenides. Acta Cryst..

[88] Tarasova N., Animitsa I., Galisheva A. (2020). Effect of doping on the local structure of new block-layered proton conductors based on BaLaInO_4_. J. Raman Spec..

[89] Tarasova N., Animitsa I., Galisheva A. (2021). Spectroscopic and transport properties of Ba- and Ti-doped BaLaInO_4_. J. Raman Spec..

[90] Shpanchenko R.V., Antipov E.V., Kovba L.M. (1993). Ba2ZrO_4_ and its hydrates. Mater. Sci. Forum.

[91] Toda K., . Kameo Y., Kurito S., Sato M. (1996). Intercalation of water in a layered perovskite compound, NaEuTiO_4_. Bull. Chem. Soc. Jpn..

[92] Chen D., Jiao X., Xu R. (1999). Hydrothernal synthesis and characterization of the layered titanates MLaTiO_4_ (M = Li, Na, K) Powders. Mater. Res. Bull..

[93] Schaak E.R., Mallouk T.E. (2001). KLnTiO_4_ (Ln = La, Nd, Sm, Eu, Gd, Dy): A New Series of Ruddlesden-Popper Phases Synthesized by Ion-Exchange of HLnTiO_4_. J. Solid State Chem..

[94] Nishimoto S., Matsuda M., Miyake M. (2005). Novel protonated and hydrated n = 1 Ruddlesden–Popper phases, H_x_Na_1−x_LaTiO_4_∙yH_2_O, formed by ion-exchange/intercalation reaction. J. Solid State Chem..

[95] Nishimoto S., Matsuda M., Harjo S., Hoshikawa A., Kamiyama T., Ishigaki T., Miyake M. (2006). Structural change in a series of pro-tonated layered perovskite compounds, HLnTiO_4_ (Ln = La, Nd and Y). J. Solid State Chem..

[96] Zvereva I.A., Silyukov O.I., Chislov M.V. (2011). Ion-Exchange Reactions in the Structure of Perovskite-like Layered Oxides: I. Proto-nation of NaNdTiO_4_ Complex Oxide. Russian. J. Solid State Chem..

[97] Kochetova N., Animitsa I., Medvedev D., Demin A., Tsiakaras P. (2016). Recent activity in the development of proton conducting oxides for high-temperature applications. RSC Adv..

[98] Tarasova N., Colomban P., Animitsa I. (2018). The short-range structure and hydration process of fluorine-substituted double perovskites based on barium-calcium niobate Ba_2_CaNbO_5.5_. J. Phys. Chem. Solids.

[99] Tarasova N., Animitsa I. (2018). Anionic doping (F^−^, Cl^−^) as the method for improving transport properties of proton-conducting perov-skites based on Ba_2_CaNbO_5.5_. Solid State Ion..

[100] Tarasova N.A., Filinkova Y.V., Animitsa I.E. (2002). Hydration and forms of oxygen-hydrogen groups in oxyfluorides Ba_2−0.5x_In_2_O_5−x_F_x_. Russ. J. Phys. Chem. A.

[101] Tarasova N., Animitsa I., Galisheva A. (2021). Effect of acceptor and donor doping on the state of protons in block-layered structures based on BaLaInO_4_. Solid State Comm..

[102] Turrillas X., Sellars A.P., Steele B.C.H. (1988). Oxygen Ion Conductivity in Selected. Ceramic Oxide Materials. Solid State Ion..

[103] Zhen Y.S., Goodenough J. (1990). Oxygen-ion conductivity in Ba_8_In_6_O_17_. Mat. Res. Bull..

[104] Poulsen F., der Puil N. (1992). Phase relations and conductivity of Sr- and La-zirconates. Solid State Ion..

[105] Navas C., Loye H.C. (1997). Conductivity studies on oxygen-deficient Ruddlesden-Popper phases. Solid State Ion..

[106] Lee D., Lee N.H. (2017). Controlling Oxygen Mobility in Ruddlesden–Popper Oxides. Materials.

[107] Chroneos A., Yildiz B., Tarancón A., Parfitt D., Kilner J.A. (2011). Oxygen diffusion in solid oxide fuel cell cathode and electrolyte ma-terials: Mechanistic insights from atomistic simulations. Energy Environ. Sci..

[108] Chroneos A., Vovk R.V., Goulatis I.L. (2010). Oxygen transport in perovskite and related oxides: A brief review. J. Alloys Compd..

[109] Kushima A., Parfitt D., Chroneos A., Yildiz B., Kilnerband J.A., Grimes R.W. (2011). Interstitialcy diffusion of oxygen in tetragonal La_2_CoO_4+δ_. Phys. Chem. Chem. Phys..

[110] Ding P., Li W., Zhao H., Wu C., Zhao L., Dong B., Wang S. (2021). Review on Ruddlesden–Popper perovskites as cathode for solid oxide fuel cells. J. Phys. Mater..

[111] Tealdi C., Ferrara C., Mustarelli P., Islam M.S. (2012). Vacancy and interstitial oxide ion migration in heavily doped La_2−x_Sr_x_CoO_4±δ_. J. Mater. Chem..

[112] Xu S., Jacobs R., Morgan D. (2018). Factors controlling oxygen interstitial diffusion in the Ruddlesden-Popper oxide La_2−x_Sr_x_NiO_4+δ_. Chem. Mater..

[113] He H., Huang X., Chen L. (2000). Sr-doped LaInO_3_ and its possible application in a single layer SOFC. Solid State Ion..

[114] Kato S., Ogasawara M., Sugai M., Nakata S. (2004). Crystal Structure and Oxide Ion Conductivity of the In-Containing K_2_NiF_4_-type Oxides. J. Ceram. Soc. Jpn..

[115] Yaguchi H., Fujii K., Yashima M. (2020). New Structure Family of Oxide-ion Conductors Based on BaGdInO_4_. J. Mater. Chem. A.

[116] Grotthuss C.J.T. (2006). Memoir on the decomposition of water and of the bodies that it holds in solution by means of galvanic electricity. Biochim. Biophys. Acta—Bioenerg..

[117] Li X., Shimada H., Ihara M. (2013). Conductivity of New Electrolyte Material Pr_1−x_M_1+x_InO_4_ (M = Ba,Sr) with Related Perovskite Structure for Solid Oxide Fuel Cells. CS Trans..

[118] Shiraiwa M., Kido T., Fujii K., Yashima M. (2021). High-temperature proton conductors based on the (110) layered perovskite BaNdScO_4_. J. Mat. Chem. A.

[119] Kreuer K.D. (2003). Proton-conducting oxides. Annu Rev Mater Res..

[120] Liu J., Ma J., Zhang Z., Qin Y., Wang Y.-J., Wang Y., Tan R., Duan X., Tian T.Z., Zhang C.H. (2021). 2021 Roadmap: Electrocatalysts for green catalytic processes. J. Phys. Mater..

[121] Chen G., Feldhoff A., Weidenkaff A., Li C., Liu S., Zhu X., Sunarso J., Huang K., Wu X., Ghoniem A.F. (2021). Roadmap on Sustainable Mixed Ionic-Electronic Conducting Membranes. Adv. Funct. Mater..

[122] Molenda J., Kupecki J., Baron R., Blesznowski M., Brus G., Brylewski T., Bucko M., Chmielowiec J., Ćwieka K., Gazda M. (2017). Status report on high temperature fuel cells in Poland—Recent advances and achievements. Int. J. Hydrog. Energy.

[123] Irvine J., Rupp J.L.M., Liu G., Xu X., Haile S.M., Qian X., Snyder A., Freer R., Ekren D., Skinner S. (2021). Roadmap on inorganic perovskites for energy applications. J. Phys. Energy.

